# GERONIMO: A tool for systematic retrieval of structural RNAs in a broad evolutionary context

**DOI:** 10.1093/gigascience/giad080

**Published:** 2023-10-17

**Authors:** Agata M Kilar, Petr Fajkus, Jiří Fajkus

**Affiliations:** Mendel Centre for Plant Genomics and Proteomics, CEITEC Masaryk University, Brno CZ-62500, Czech Republic; Laboratory of Functional Genomics and Proteomics, NCBR, Faculty of Science, Masaryk University, Brno CZ-61137, Czech Republic; Mendel Centre for Plant Genomics and Proteomics, CEITEC Masaryk University, Brno CZ-62500, Czech Republic; Department of Cell Biology and Radiobiology, Institute of Biophysics of the Czech Academy of Sciences, Brno CZ-61265, Czech Republic; Mendel Centre for Plant Genomics and Proteomics, CEITEC Masaryk University, Brno CZ-62500, Czech Republic; Laboratory of Functional Genomics and Proteomics, NCBR, Faculty of Science, Masaryk University, Brno CZ-61137, Czech Republic; Department of Cell Biology and Radiobiology, Institute of Biophysics of the Czech Academy of Sciences, Brno CZ-61265, Czech Republic

**Keywords:** sequence homology searches, evolution, high-throughput pipeline, Snakemake

## Abstract

**Background:**

While web-based tools such as BLAST have made identifying conserved gene homologs appear easy, genes with variable sequences pose significant challenges. Functionally important noncoding RNAs (ncRNA) often show low sequence conservation due to genetic variations, including insertions and deletions. Rather than conserved sequences, these RNAs possess highly conserved structural features across a broad phylogenetic range. Such features can be identified using the covariance models approach, which combines sequence alignment with a secondary RNA structure consensus. However, running standard implementation of that approach (Infernal) requires advanced bioinformatics knowledge compared to user-friendly web services like BLAST. The issue is partially addressed by RNAcentral, which can be used to search for homologs across a broad range of ncRNA sequence collections from diverse organisms but not across the genome assemblies.

**Results:**

Here, we present GERONIMO, which conducts evolutionary searches across hundreds of genomes in a fully automated way. It provides results extended with taxonomy context, as summary tables and visualizations, to facilitate analysis for user convenience. Additionally, GERONIMO supplements homologous sequences with genomic regions to analyze promoter motifs or gene collinearity, enhancing the validation of results.

**Conclusion:**

GERONIMO, built using Snakemake, has undergone extensive testing on hundreds of genomes, establishing itself as a valuable tool in the identification of ncRNA homologs across diverse taxonomic groups. Consequently, GERONIMO facilitates the investigation of the evolutionary patterns of functionally significant ncRNA players, whose understanding has previously been limited to individual organisms and close relatives.

## Introduction

The characterization of noncoding RNAs (ncRNAs) has been an ongoing and exciting area of research since the 1950s. Research has shown that ncRNAs play a vital role in various cellular processes, such as providing a decoding scaffold for transfer RNA (tRNA) [1] and building components of the translation machinery complex, such as ribosomal RNAs [2]. Additionally, small nuclear RNAs (snRNAs) are involved in splicing events as constituent components of the spliceosome; examples of these include U1, U2, U4, U5, and U6 (reviewed in [3]). Subsequently, the roles of ncRNAs have been found to extend beyond translation, ribosomal activities, and splicing events, encompassing diverse functions (reviewed in [4]). Despite much progress in understanding the role and function of ncRNAs, there is still much to discover regarding new ncRNA families and their functions [5].

Classes of ncRNAs significantly vary in terms of sequence length, functions, biological occurrence, structure, and distribution across species [6]. A notable example of ncRNA with a highly heterogeneous sequence is telomerase RNA (TR), which acts as a template for the extension of chromosome ends by the telomerase complex. The lengths of TRs vary considerably, ranging from 159 nucleotides in *Tetrahymena* to around 1,200 nucleotides in yeast, *Saccharomyces cerevisiae* [7]. The extremely high diversity among TRs in terms of their lengths, sequences, and biosynthesis pathways considerably complicates their identification. Consequently, TRs have been elusive in the plant kingdom for over 30 years compared to those in other organisms, such as humans or yeast [8, 9].

When performing sequence similarity searches across different species, whether evolutionarily related or not, BLAST is a popular tool and is often the first choice due to its widespread use [10]. However, BLAST is limited in identifying novel ncRNAs, as they differ from protein-coding genes and do not necessarily maintain a preserved reading frame. Thus, ncRNAs often contain small insertions or deletions that disrupt the exact seed match on which BLAST search relies. Moreover, many ncRNAs are often too short for an effective BLAST search. The limitations of sequence homology search methods for identifying novel ncRNA sequences have been addressed by the emergence of advanced computational methods such as Infernal [11]. Infernal generates probabilistic profiles of aligned ncRNA sequences and their secondary structure consensus, known as covariance models. A statistically significant covariation (correlated variation) suggests that 2 nucleotides in the alignment form a base pair that is important to the function of the RNA because there has been selective pressure to maintain it through compensatory base pair substitutions. A homology search performed with Infernal produced results as good as a provided covariance model. Based on this, optimization strategies for alignment composition were proposed [12]. Furthermore, Infernal has enabled the creation of the Rfam database, which contains over 3,400 reliable alignments for structural RNA families [13] and allows for the identification of homologous ncRNA sequences in related species with the aid of many precalculated covariance models.

In cases where existing covariance models cannot initiate homology sequence searches, it is necessary to create a new set of alignments to identify novel ncRNAs. In this process, knowledge of RNA secondary structure can be particularly helpful. Advances in affordable and precise RNA sequencing methods have significantly improved our understanding of RNA function [14]. However, a “sequence-structure gap” has emerged, where many RNA molecules have been sequenced, but their structures remain unknown. To address this gap, computational tools have been proposed that aim to predict RNA secondary structure. These tools include those based on minimum free-energy calculations, such as RNAstructure or RNAfold [15, 16], as well as those that integrate thermodynamic parameters with comparative analysis based on alignments, such as RNAalifold or Turbofold II [17, 18]. The main limitations of these methods are accumulating errors in energy calculations and the tendency to “overfold” RNA structures [19]. Several machine learning methods are now being used to explore RNA structure [19]. However, their learning process is primarily based on existing databases of RNA structure and may be biased toward certain RNA types (e.g., those used in the small training set during the learning process). Moreover, their learning process often lacks biophysical or evolutionary significance, making generalizing across different RNA families challenging [20].

RNAcentral, a web-based platform, has improved the accessibility of homology searches [21]. This is primarily due to its ability to allow for searches across a vast array of ncRNA databases simultaneously, using simple fasta queries. However, RNAcentral is not equipped to facilitate searches within whole-genome data. In addition to this limitation, the platform also poses other challenges that can render the analysis process cumbersome. One of the principal obstacles is the indistinct evolutionary context, which necessitates time-consuming analysis of results. Often, among the vast number of results returned, only the name of the organism is given, without any information regarding the broader taxonomy, making it difficult to draw meaningful conclusions. Another challenge is the difficulty in manipulating the analysis range. The search can return an overwhelming number of results, which can be unhelpful, especially when looking for homologies in specific evolutionary lineages, particularly in the absence of a taxonomic context. The search results are presented as a text file containing the aligned sequences, with limited options for data filtering. One possible approach is to sort the results based on the similarity or significance level between the query and the targeted sequence. However, this process can be time-consuming and requires manual inspection of the results. Lastly, lacking information on the neighboring genomic region can hinder the validation process. Access to the genomic region around the homologous sequence could enable researchers to search for specific DNA motifs, for example, within the promoter region.

In this work, we present GERONIMO (GEnomic RNA hOmology aNd evolutIonary MOdeling), a bioinformatics pipeline that uses the Snakemake framework to conduct high-throughput homology searches of ncRNA genes using covariance models on any evolutionary scale. GERONIMO allows users to specify *what to search for* by providing a covariance model or multiple alignments in Stockholm format and *where to search* by defining a targeted database that can be easily set up at NCBI’s database service and range in scale from order through family, clade to phylum or kingdom. The pipeline can be run with one command, making it easy to use without additional user intervention. After the homology search is complete, GERONIMO generates comprehensive and accessible tables that present all essential information regarding the query and target sequence similarity levels. These tables are enriched with a broad taxonomy context, which enables effective data filtering and minimizes false-positive results through the assessment of evolutionary relationships. Furthermore, GERONIMO provides visual representations of the table results, which makes it easier to draw holistic conclusions and refine novel covariance models. In addition to these advancements, GERONIMO provides each significant candidate sequence with extended genomic regions, which might be searchable for specific DNA motifs, becoming handy in results validation. This makes GERONIMO a recipe for conducting routine homology searches on any taxonomy scale supported with results overview, enabling novel ncRNA exploration more accessible with an evolutionary context.

## Implementation

### Pipeline overview

GERONIMO (RRID: SCR_023899) is a high-throughput bioinformatics pipeline that uses covariance models based on sequence alignment and secondary structure consensus to search for unknown genetic elements. It is built with Snakemake, a reproducible workflow management tool that operates on multiple computational platforms, like Windows 10 and Ubuntu. The pipeline assesses dependencies during its initial run to ensure compatibility with various platforms.

One major advantage of GERONIMO is its high level of parallelization, which accelerates the analysis process by conducting simultaneous analyses of single genomes and covariance models. With access to 24 CPUs, for example, GERONIMO can analyze at least 6 genomes simultaneously. Snakemake’s parallelization module optimizes the distribution of computational resources among all pipeline components, with most components requiring only 1 CPU.

**Figure 1:**
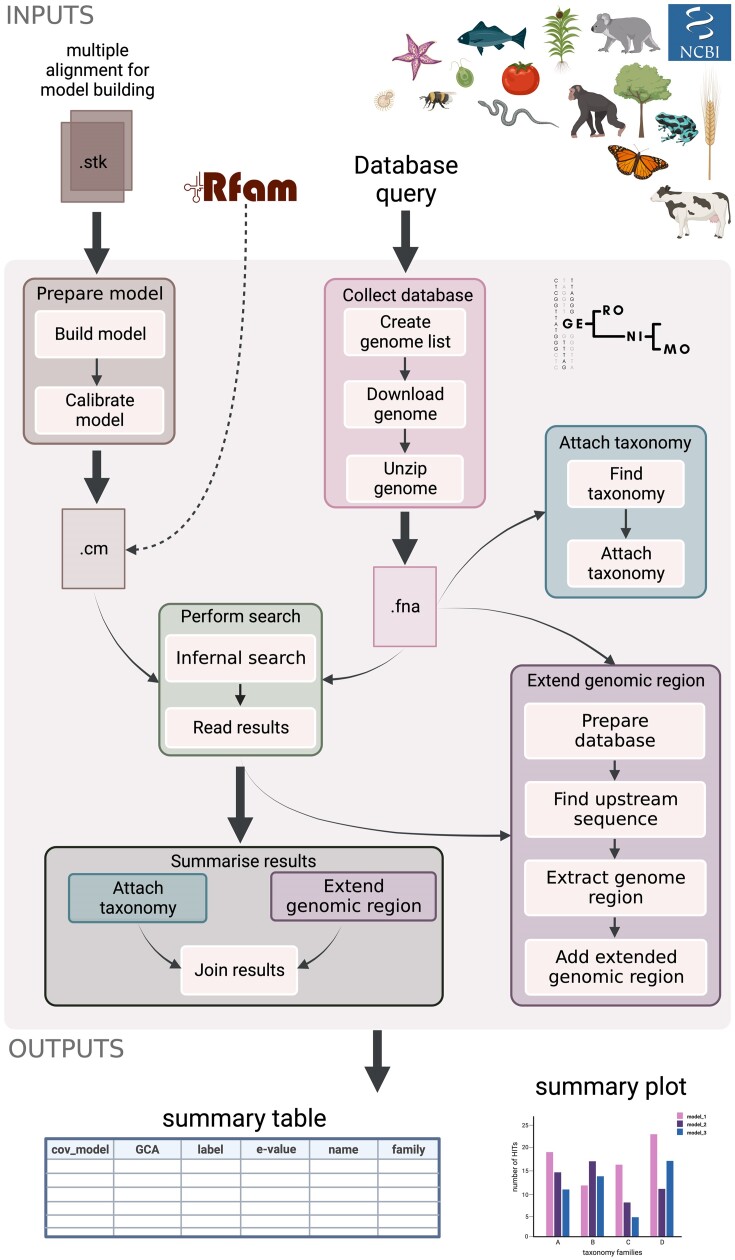
The GERONIMO workflow representing the pipeline components.

The schematic view of GERONIMO is shown in Fig. 1.

### Inputs characterization

GERONIMO accepts an NCBI database query as input, which allows for identifying the genomes used in the analysis. The database query can be easily modified at NCBI to ensure control over the desired dataset.

GERONIMO requires a covariance model to conduct evolutionary searches across the genome database. The covariance model can be obtained from the *Rfam* database [13] or built with GERONIMO using multiple sequence alignments and a consensus structure. These alignments can be generated through *RNAalifold* web services [17].

### Database collection

The specified database query provided by the user is used to download the relevant genomes from the *NCBI Assembly database* [22]. The *Entrez* (NCBI) tool [23] is used to generate a list of all genomes identified by the database query, which is exported to the “list_of_genomes.txt” file in the main GERONIMO folder. The links to the specific genomes are then sent to the *rsync* BASH utility, which downloads each genome and checks its integrity using the *md5 hash*. The downloaded genomes are in *.fna.gz* format. Thus, they must be unzipped for further analysis using the *gunzip* BASH utility.

### Covariance model preparation (optional)

GERONIMO allows creating a custom model using multiple sequence alignments with a secondary consensus structure in *.stk* format. The model is built using the incorporated Infernal module and the *cmbuilt* tool, followed by calibration with the *cmcalibrate* tool [11]. To optimize the analysis flow, the user can allocate the desired number of CPUs for the calibration process in the config.yaml file, considering its resource-intensive nature.

### Evolutionary search

The *cmsearch* (Infernal) tool conducts the search by comparing the covariance model to the genome sequence to identify similarities. Detailed information on the operation and output structures of Infernal can be found in the tool manual [11].

*Infernal* produces 3 output files in space-delimited text format, which can be found in the *GERONIMO/results/infernal_raw* folder, organized by model and specific genome. The results from the evolutionary search are categorized by *Infernal* based on the statistical significance of the level of similarity—*hit*. A hit is considered significant when its e-value is less than 0.01 and is marked with a “!” symbol. A value between 0.01 and 10 is regarded as a potentially compelling finding and is marked with a “?” symbol. GERONIMO adheres to this convention throughout the analysis, labeling significant hits as “HIT” and potential findings as “MAYBE.” Additionally, any value greater than 10 is classified as a “NO HIT” by GERONIMO.

### Reading *Infernal* results

The output structure of Infernal’s files makes it challenging to concatenate the overall results, especially when conducting extensive analysis with multiple models and hundreds of genomes. To address this issue, a custom script was developed to reformat the output structure and present it in a more manageable table format. The script written in *R* [24], using the *tidyverse* package [25], handles the space-delimited nature of Infernal’s output and converts it to an accessible *.csv* format.

### Attaching taxonomy context

One limitation of working with genome assemblies downloaded from the NCBI database is that they are often described only with a GCA id, which can complicate results analysis and lack informative context. GERONIMO addresses this issue by providing a broad taxonomy context by extracting taxonomic information such as the organism name, family, order, class, and phylum. This is achieved using a custom script in *R* [24] that uses the *rentrez* utilities [26] to extract taxonomy information from the NCBI taxonomy database based on the unique GCA id. The taxonomy information is then restructured into a table format, allowing it to be easily joined with the processed Infernal results. The individual taxonomy information can be found in the *GERONIMO/taxonomy folder*, named with the GCA id.

### Extending the genomic region

GERONIMO offers extended genomic context for each significant hit by extracting the upstream and downstream region using the *blastcmd* tool preceded by the *makeblastdb* tool, both of which are part of the BLAST+ package [27]. This functionality allows searching of specific DNA motifs whose presence or absence can aid in hit validation.

If a significant hit is identified in the genome with the covariance model, the coordinates of the hit are adjusted by adding or subtracting a specified length of the extracted region, depending on the location of the hit relative to the forward or reverse strand. This length can be specified in the config.yaml file under the *extract_genomic_region-length* parameter. We tested using a distance of approximately 200 nucleotides in all runs. It is important to note that genome assemblies are composed of contigs, and in some cases, the hit may be located near the beginning or end of a contig. If the region of interest exceeds the contig range, the upstream sequence cannot be extracted and requires manual curation.

### Results summarization

In the final module of GERONIMO, all individual partial results are concatenated into the *summary_table.xlsx* file, which is the main output of the pipeline. Joining the results is achieved using the *cat* BASH utility. The aggregated results are then passed to a custom *R* script, which adds column names and arranges the results into a widely used Excel table format.

To facilitate the analysis of the results, a separate “summary_table_models.xlsx” table is generated, which separates the pipeline results into spreadsheets according to the covariance models applied. This feature proves especially useful when evaluating the performance of multiple models in a single run.

Based on the summary table, GERONIMO produces summary visualizations in 2 plots. The first plot is a bar chart showing the number of genomes per family in which at least 1 significant HIT was found. The second plot presents the significance of the HITs in a heatmap, where all individual genomes are grouped into taxonomy families. The value represents the most significant HIT from each particular genome.

## GERONIMO applicability

The tool was designed to address the limitations of commonly used tools for performing sequence homology searches in an evolutionary context across multiple taxonomy levels, ranging from families to kingdoms. The tool was developed in collaboration with biology experts to ensure applicability and can be easily used with a single command line. The GERONIMO website, hosted on GitHub, includes comprehensive guidelines on tool requirements, installation, usage, and potential pitfalls described in the “Questions and Answers” section.

### Standard analysis

GERONIMO is particularly useful in analyses that require testing multiple covariance models, as it provides parallelization utilities and visual output, facilitating a better interpretation of results. On a local machine equipped with an Intel Core i5-10400 CPU 2.90 GHz and 16 GB RAM, a standard GERONIMO run that includes 8 covariance models of highly conserved gene families, such as snRNAs, with around 50 genome assemblies of the Rhodophyta phylum, takes approximately 2 to 3 hours.

### Expanding the evolutionary context

By providing a new database query, the scope of the analysis can be broadened. GERONIMO will generate additional results for previously used covariance models, integrate them with earlier results, and present updated summary tables and visualizations. This is an efficient process as genomes that have already been downloaded do not need to be re-downloaded; only newly added genomes will be obtained from the updated database query. This feature is important in reducing the overall time required for complex homology searches and allowing for them to be easily updated with rapidly expanding newly available data.

### Incorporating different types of models

GERONIMO offers a significant advantage in that it enables easy integration of new models into a preexisting analysis. The tool will perform additional homology searches and combine them with previous analyses using the same database, thereby saving time and resources as existing results are not recalculated. This feature makes GERONIMO an efficient solution for adding and comparing new models.

### Model training

GERONIMO offers the capability to create novel covariance models and optimize existing ones. The tool provides the option to build new covariance models and test their performance compared to original models, including situations where no covariance models are available in the Rfam database. This feature allows researchers to create and fine-tune models to meet their specific research needs.

## Testing

### Validation strategy

The performance of GERONIMO was validated by comparing it to the widely used BLASTn tool. The transcribed region of *Arabidopsis thaliana* telomerase RNA (AtTR) was used as the query sequence for the BLASTn search, performed with standard parameters. Meanwhile, an alignment file with a secondary structure consensus was created from the same sequence using the RNAalifold web services [17]. The input files used in the comparison are detailed in Supplemental Material 1. As the dataset, we used representative genome assemblies of Tracheophytes available in the NCBI database [22].

### Results of GERONIMO with BLASTn and RNAcentral comparison

**Figure 2:**
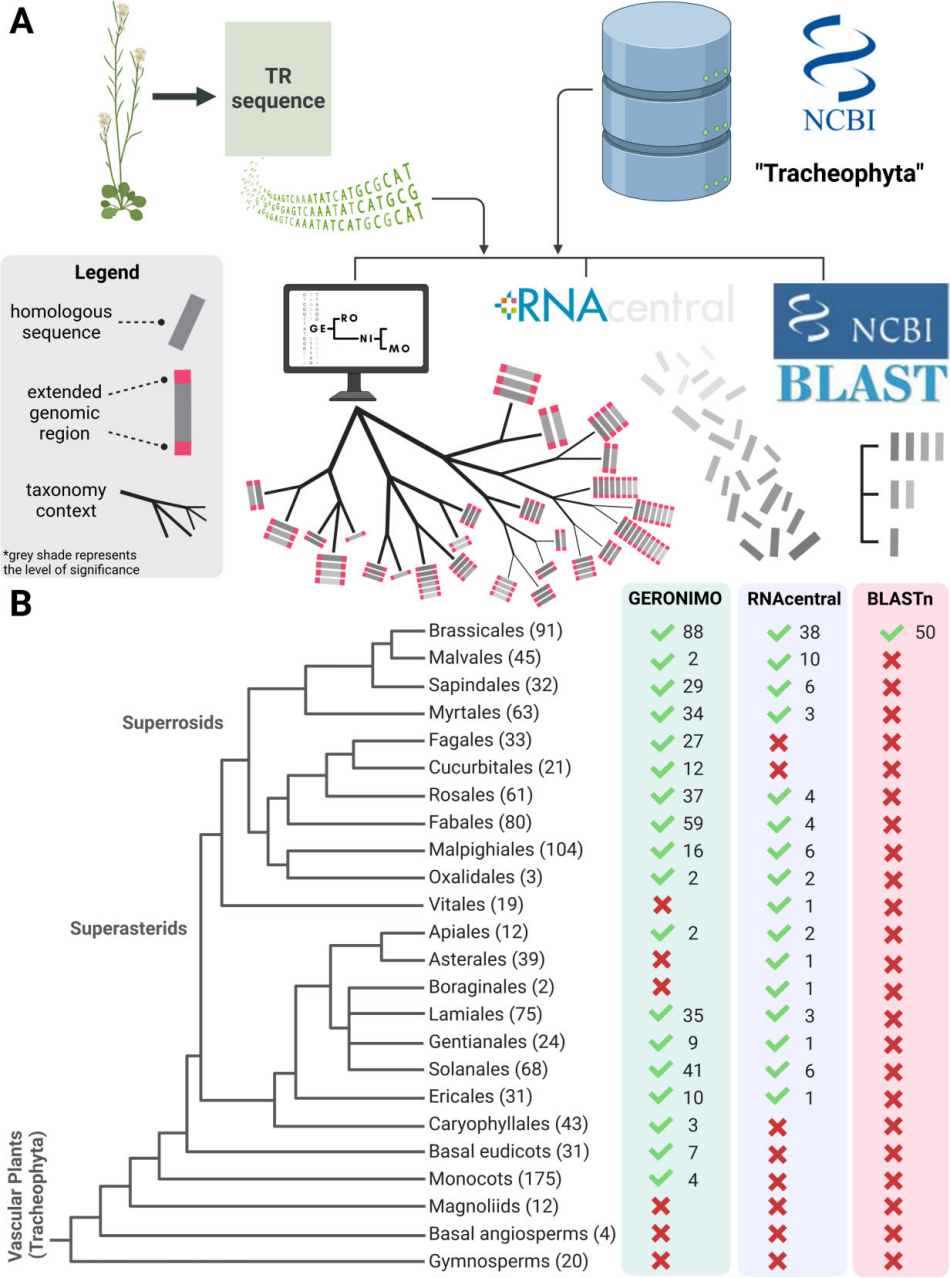
Overview of the strategy for comparing the performance of GERONIMO, RNAcentral, and BLASTn and their results. (A) The comparison process involved using the AtTR sequence as a query in a targeted database of 1,101 genome assemblies available in the NCBI database to find homologous candidates. The use of GERONIMO yielded results that were supported by extended genomic regions and broad taxonomic contexts. In contrast, RNAcentral only provided homologous sequences, while BLASTn offered homologous sequences with plain evolutionary context. (B) A simplified phylogenetic tree of the Tracheophyta clade was used, including the extended orders of Superasterids and Superrosids, along with the number of representative genome assemblies used in the comparison. The branch lengths do not express real-time scales. The green bar represents the performance of GERONIMO, the violet bar represents the performance of RNAcentral, and the red bar represents the performance of BLASTn, with the number of genomes in which TR candidates were identified (marked with a green tick). The red cross indicates a failure to identify TR within a given order.

GERONIMO’s performance in homology sequence searches was compared to that of available services such as BLASTn and RNAcentral. The TR gene of *Arabidopsis thaliana* from the Brassicales order was our search query, and we selected 1,101 representative genome assemblies from Tracheophytes available in the NCBI database as our reference target database. The comparison strategy is presented in Fig. 2A, and the results are available in Supplementary Table S1.

BLASTn identified similar sequences in approximately 5% of the targeted genomes. Specifically, it provided homologous sequences in 55% of genome assemblies belonging to the same order as the query (Brassicales). However, it failed to detect significant homologs even in closely related orders, such as Malvales or Sapindales (see Fig. 2B).

The homology search conducted using RNAcentral produced a large number of results. However, these were limited solely to preexisting annotated ncRNA collections. The search identified 38 significant sequences within the Brassicales order from 27 unique organisms, of which 24 were classified as TR. In addition, RNAcentral detected 352 similar sequences in 100 unique organisms within Tracheophyta, of which 46 were identified as TR, and most were classified as long ncRNA. The results were downloaded in FASTA format and manually filtered to account for evolutionary context. However, RNAcentral did not provide the secondary structure for the given query and reported that it did not match any Rfam family.

GERONIMO detected homologous sequences in 97% of the Brassicales genomes tested and identified 29 TRs in the neighboring Sapindales order and 2 TRs in the Malvales order (Fig. 2B). Additionally, GERONIMO detected 306 TRs in the Superrosids clade and 107 TRs in the large Superasterids clade. Notably, without any optimization, GERONIMO could detect homologous sequences across distant clades, including basal eudicots and even monocots. To confirm the reliability of the GERONIMO results, we analyzed the presence of promoter-specific motifs in the extended upstream genomic region.

Overall, all the methods tested facilitate the sorting and validation of hits based on their significance, as measured by the e-value. However, when dealing with evolutionary highly divergent ncRNAs (e.g., TRs), applying a significance threshold often results in either no hits or hits with controversial homology (i.e., high e-value). Given the challenges posed by the low conservation of ncRNAs, leveraging the surrounding genomic context offered by GERONIMO can be particularly useful in validating hits. A good example of this validation is the analysis of conserved promoter elements or gene collinearity, which may be evolutionarily more conserved than the ncRNA sequence itself.

## Discussion

Discovering novel ncRNA sequences through evolutionary homology might seem straightforward initially, then turn out to be challenging due to the relatively short length of ncRNAs, often 100 nucleotides or less, and the lack of sequence conservation [28]. Although comparative data analyses can be performed among closely related species (using BLASTn) or across the range of databases (using RNAcentral), they often lack intuitive outputs that facilitate drawing conclusions. While BLAST provides simplified taxonomic overviews, it is not well suited for ncRNA searches, which limits the taxonomic range that can be explored. Nevertheless, BLAST can still be helpful for preliminary searches due to its user-friendly interface and ease of use. The RNAcentral Consortium is a crucial advancement in ncRNA research. It offers a consolidated platform that allows access to data from several independent ncRNA resources, including Rfam, NCBI, and ENA. The platform also supports the use of various homology search tools like nhammer, which enhances the identification of ncRNAs [21]. Additionally, it provides complementary tools like R2DT, which generates secondary structure diagrams, thus improving the process of studying ncRNAs [29].

The discovery of novel ncRNAs relies on evidence of evolutionary conservation of RNA sequence and structure. Currently, structural prediction alone is insufficient evidence of a conserved structure, and false discoveries can arise due to artifacts introduced by misalignments in the analysis [30]. Pseudogenes pose another challenge, as they exhibit sequence and structure conservation but do not *function* as ncRNAs. The presence of pseudogenes in alignments of structural ncRNAs can dilute the covariation signal, resulting in an overall increase in power but a decrease in covariation at base-paired positions [31].

Despite the challenges associated with RNA structure analysis, Infernal has been successfully used to characterize a large number of ncRNAs in the Rfam database [32]. Infernal has also been applied for the identification of novel TRs in Metazoa [33] and integrated as a module in other bioinformatics tools, including MITOS for *de novo* annotation of mitochondrial genomes [34], tRNAscan for identifying tRNAs [35, 36], and FASTAptameR 2.0 for simple motif discovery [37]. Recent advances in the field have enabled the combination of Infernal with R-scape and the CaCoFold algorithm, identifying 17 novel structurally conserved ncRNA candidates across 5 fungal genomes [38].

As Dobzhansky famously stated, “Nothing in Biology Makes Sense Except in the Light of Evolution” [39]. This statement holds true for successful homology searches, as the lack of a broad evolutionary context can hinder progress. This is especially evident for independently transcribed RNAs, where the genomic context is often more conserved than the RNA sequence itself. In addition, ncRNA genes frequently reside in homologous intergenic or intronic regions, even across large evolutionary distances [12]. Moreover, GERONIMO offers a unique feature that extends the homologous sequence with neighboring genomic context, providing the opportunity to search, for example, promoter-specific motifs. This feature can effectively exclude false-positive candidates and detect pseudogenes, thereby strengthening the analysis. The availability of information on the neighboring genomic region around the homologous sequence supports the validity of results. GERONIMO was developed with a focus on the broad evolutionary context, building on our previous research on the characterization of novel TRs. This effort led to the discovery of 328 novel TRs in early diverging plants and insects, demonstrating the effectiveness of the approach [40, 41].

To date, hundreds of functionally important ncRNAs have been characterized as crucial players in various biological processes, such as adaptation to stress, chromatin remodeling, and diverse developmental pathways. Nevertheless, the knowledge about these ncRNAs often remains confined to specific model organisms. It is essential to recognize that a significant portion of these finely tuned ncRNA machineries did not emerge by chance but rather underwent millions of years of evolution. From this perspective, GERONIMO holds the potential to offer valuable insights into ncRNA precursors present in early diverged organisms. By analyzing the evolutionary history of these molecules across diverse taxa, GERONIMO can shed light on their ancient origins and elucidate the intricate processes that have shaped their functionality over time. This information can significantly contribute to our understanding of the broader role and conservation of ncRNAs across the tree of life, enhancing our knowledge of the fundamental biological mechanisms underlying life’s complexity.

GERONIMO is a unique tool that enables routine searches for novel ncRNA sequences in any evolutionary context, providing quickly analyzable tables and plots of the results. The table format allows for filtering based on the significance of homologous sequences and taxonomy information, which can help identify the best candidates within a given family, order, or clade. The summary visualization of the homology search provides insight into the overall results of the analysis, such as the number of candidates identified within a given family, which can aid in identifying the best candidates for better alignment composition and ultimately lead to improved performance of covariance models.

Finally, GERONIMO is available on various platforms, including Windows 10 and Linux, and can take advantage of external services for extensive computational power.

## Availability of Source Code and Requirements

Project name: GERONIMO

Project homepage: https://github.com/amkilar/GERONIMO.git

Operating system(s): Linux, <Windows 10 (WSL)

Programming language: Python3, bash, R

Other requirements: <conda 23.3.1

License: MIT license

RRID: SCR_023899

bio.tools ID :GERONIMO

GERONIMO is also registered on workflowhub.eu [42].

## Supplementary Material

giad080_GIGA-D-23-00157_Original_Submission

giad080_GIGA-D-23-00157_Revision_1

giad080_Response_to_Reviewer_Comments_Original_Submission

giad080_Reviewer_1_Report_Original_Submission Dr Chao-Chung Kuo -- 7/18/2023 Reviewed

giad080_Reviewer_2_Report_Original_Submission Blake Sweeney -- 7/10/2023 Reviewed

giad080_Reviewer_2_Report_Revision_1 Blake Sweeney -- 8/10/2023 Reviewed

giad080_Supplemental_Files

## Data Availability

Supporting data sets for this article are available via Figshare [43] and GigaDB [44].

## Abbreviations

AtTR: Arabidopsis thaliana telomerase RNA; BLAST: Basic Local Alignment Search Tool; GERONIMO: GEnomic RNA hOmology aNd evolutIonary Modeling; NCBI: The National Center for Biotechnology Information; ncRNA: noncoding RNA; snRNA: small nuclear RNA; TR: telomerase RNA; tRNA: transfer RNA.

## Additional Files

**Supplementary Table S1**. Comparison of the results obtained in the validation process.

**Supplementary Material 1**. Input queries for the validation process.
