# Supplementary material for: GERONIMO: A tool for systematic retrieval of structural RNAs in a broad evolutionary context
Source: Gigascience. 2023 Oct 17;12:giad080. doi: 10.1093/gigascience/giad080 (PMC10580375; doi:10.1093/gigascience/giad080)
Supplement: giad080_GIGA-D-23-00157_Revision_1 [file giad080_giga-d-23-00157_revision_1.pdf]

GigaScience

GERONIMO: A Tool for Systematic Retrieval of Structural RNAs in Broad Evolutionary Context

--Manuscript Draft--

Manuscript Number:	GIGA-D-23-00157R1
Full Title:	GERONIMO: A Tool for Systematic Retrieval of Structural RNAs in Broad Evolutionary Context
Article Type:	Technical Note
Funding Information:	International Visegrad Fund (52210685) — Dr. Agata Magdalena Kilar<br>Grantová Agentura České Republiky (20-01331X) — Prof. Jiri Fajkus
Abstract:	Background<br><br>While web-based tools such as BLAST have made identifying conserved gene homologs appear easy, genes with variable sequences pose significant challenges. Functionally important non-coding RNAs often show low sequence conservation due to genetic variations, including insertions and deletions. Rather than conserved sequences, these RNAs possess highly conserved structural features across a broad phylogenetic range. Such features can be identified using the covariance models approach, which combines sequence alignment with a secondary RNA structure consensus. However, running standard implementation of that approach (Infernal) requires advanced bioinformatics knowledge compared to user-friendly web services like BLAST. The issue is partially addressed by RNAcentral, which can be used to search for homologs across a broad range of ncRNA sequence collections from diverse organisms but not across the genome assemblies.<br><br>Results<br><br>Here, we present GERONIMO, which conducts evolutionary searches across hundreds of genomes in a fully-automated way. It provides results extended with taxonomy context, as summary tables and visualisations to facilitate analysis for user convenience. Additionally, GERONIMO supplements homologous sequences with genomic regions to analyse promoter motifs or gene collinearity, enhancing the validation of results.<br><br>Conclusion<br><br>GERONIMO, built using Snakemake, has undergone extensive testing on hundreds of genomes, establishing itself as a valuable tool in the identification of non-coding RNA homologs across diverse taxonomic groups. Consequently, GERONIMO facilitates the investigation of the evolutionary patterns of functionally significant ncRNA players, whose understanding has previously been limited to individual organisms and close relatives.
Corresponding Author:	Jiri Fajkus<br>Masaryk University: Masarykova Univerzita<br>Brno, CZECH REPUBLIC
Corresponding Author Secondary Information:	
Corresponding Author's Institution:	Masaryk University: Masarykova Univerzita
Corresponding Author's Secondary Institution:	
First Author:	Agata Magdalena Kilar
First Author Secondary Information:	
Order of Authors:	Agata Magdalena Kilar

Powered by Editorial Manager® and ProduXion Manager® from Aries Systems Corporation

	Petr Fajkus
	Jiri Fajkus
**Order of Authors Secondary Information:**	
**Response to Reviewers:**	Dear Editor,<br>Thank you very much for communicating our MS to qualified reviewers. We are pleased to submit the minor revision addressing all your and reviewers' comments. Please, see below our responses:<br><br>EDITOR:<br><br>- In addition to the reviewer's reports, I'd like to mention that submissions presenting new software should discuss and compare similar, existing tools - I see you compare to BLASTn and RNAcentral, but if you are aware of any other publicly available tools that are similar or related to GERONIMO, please make sure to mention and compare them in the revised submission.<br><br>RESPONSE: Thank you for your thoughtful feedback on our manuscript. We are aware of the Evolinc II pipeline developed by A. Nelson (https://www.ncbi.nlm.nih.gov/pmc/articles/PMC5422434/), and we acknowledge its relevance as another tool aimed at identifying evolutionarily conserved ncRNAs using RNAseq data and genome assemblies. However, after carefully assessing Evolinc II, we found that the mutual comparisons of lincRNAs are BLAST-based, similar to our comparison with BLASTn. Therefore, we believe that a direct comparison with Evolinc II may not bring substantial new insights to our manuscript.<br><br>Furthermore, we are also aware that nhmmer can be employed for similar searches against genomes, but we had already implemented and utilized it in our comparison, including in the assessment against RNAcentral.<br><br>We firmly believe that the existing comparisons with RNAcentral and BLASTn sufficiently support the claims made in our submission. Nonetheless, we are grateful for your suggestion and appreciate your interest in further validation. If you have any other questions or concerns, please do not hesitate to share them with us. We are committed to improving the manuscript based on valuable feedback like yours.<br><br>- Please also carefully read our instructions for authors for the "Technical Note" article type and make sure you follow the formatting guidelines (order and headings of sections etc.)<br><br>RESPONSE: We have carefully reviewed the instructions for authors regarding the "Technical Note" article type and have made the necessary changes to ensure that our manuscript adheres to the formatting guidelines, including the correct order and headings of sections.<br><br>- In addition, please register your new software application in the bio.tools and SciCrunch.org databases to receive RRID (Research Resource Identification Initiative ID) and biotoolsID identifiers, and include these in your manuscript (in the "code availability" section). Computational workflows should be registered in workflowhub.eu and the DOIs cited in the relevant places in the manuscript. These will facilitate tracking, reproducibility and re-use of your tool.<br><br>RESPONSE: We acknowledge the importance of providing RRID and biotoolsID identifiers for our tool and ensuring that computational workflows are registered in appropriate databases like workflowhub.eu. We have taken immediate action and successfully registered GERONIMO in both bio.tools and SciCrunch.org databases, obtaining the necessary RRID and biotoolsID identifiers (SCR_023899 and biotools:GERONIMO, respectively). Additionally, we have also registered our computational workflows on workflowhub.eu and included the relevant DOIs (https://doi.org/10.48546/workflowhub.workflow.547.1) in the manuscript, as you suggested.<br><br>REVIEWER #1:

No questions or suggestions.

REVIEWER #2:

- The authors use a value of 0.05 as the E value cutoff for 'true' hits and suggest it is infernal's standard value. However, the infernal manual recommends 0.01, http://eddylab.org/infernal/Userguide.pdf page 20. Was this difference intentional?

RESPONSE: Upon carefully considering your comments, we have identified an unintentional mistake in our study regarding the e-value cutoff used for identifying 'true' hits.

You are correct in pointing out that we used a value of 0.05 as the e-value cutoff for 'true' hits, while the infernal manual recommends a more stringent value of 0.01 (http://eddylab.org/infernal/Userguide.pdf page 20). We apologize for this oversight in our initial submission.

We want to assure you that this difference in the e-value cutoff was not intentional, and we recognize the significance of using the appropriate threshold to ensure the accuracy and reliability of our findings. As soon as we became aware of this mistake through your review, we took immediate action to rectify it.

We have now revised the manuscript to reflect the correct e-value cutoff of 0.01 in all relevant sections.

- Additionally, infernal itself indicates if something is a true vs false hit using '!' or '?' in the output. Are the authors using infernal's filters or their own to select hits?

RESPONSE: In our study, we have indeed utilized infernal's '!' and '?' symbols for this purpose. However, to enhance clarity and avoid any confusion, we have translated these symbols into "HIT" and "MAYBE" in the manuscript. This modification has been made in the "Methods" section to ensure a clear representation of our approach.

We hope that this clarification addresses your concern, and we thank you for helping us improve the clarity and accuracy of our research.

- It may be necessary to use the -Z option of cmsearch to produce accurate E values. If the genome is split across several files then it is required to ensure E values are accurate. If the genome is in one file then it is not needed as cmsearch will compute it correctly. This reviewer did not examine the pipeline closely enough to determine if the genomes being searched are in a single file.

RESPONSE: In our pipeline, we perform searches on whole genomes represented as single files using Infernal. As such, we did not utilize the -Z option in the cmsearch.

It is essential to clarify that our parallelization process does not involve splitting the genome; instead, it distributes the entire genomes among the available CPUs for efficient processing.

- This reviewer isn't terribly familiar with conda environments, but the yaml files it uses appear to mention paths specific to one of the authors computers. Is this expected?

RESPONSE: You are absolutely right; we apologize for any confusion caused by the paths specified in the conda environment yaml files. Rest assured, before finalizing the manuscript, we extensively tested our software, GERONIMO, on multiple systems, not just the one with the specific path mentioned.

The results of our testing were positive, and GERONIMO performed as expected on various setups, including those without any specific paths mentioned in the yaml files. We have now made the necessary adjustments to ensure that the paths are generalized and applicable across different systems.

- There are some small cleanups that could be done to the produced figures. For

Powered by Editorial Manager® and ProduXion Manager® from Aries Systems Corporation

	example, the facet names in plot Hits_distribution_across_families, should be the model not prefixed with cov_. The green color is unclear in plots/Hits_distribution_heatmap. It is not labelled in the figure but should be. In general the figures could use some polish to be more approachable to new users.<br><br>RESPONSE: We have made the necessary adjustments to address your comments. Specifically, in the plot "Hits_distribution_across_families," we have updated the facet names to display the model without the "cov_model_" prefix, as you suggested. Additionally, in the "Hits_distribution_heatmap" plot, we have now changed the green color to grey to enhance clarity.<br><br>Furthermore, we have taken your feedback into consideration for all the figures, and we have applied touch-ups to improve their overall presentation and make them more approachable for new users.<br><br>- The images in their very comprehensive README file are broken.<br><br>RESPONSE: We would like to express our gratitude for bringing to our attention the issue with the images in our comprehensive README file.<br><br>We are pleased to inform you that we have promptly addressed this concern and fixed the broken images. The README file now displays all the images correctly, ensuring that users can access the necessary information seamlessly.<br><br>- 'The pipeline can be run with one command line' should be 'The pipeline can be run with one command'.<br><br>RESPONSE: We acknowledge the correction you pointed out regarding the statement, "The pipeline can be run with one command line." We have now revised it to say, "The pipeline can be run with one command."
**Additional Information:**	
**Question**	**Response**
Are you submitting this manuscript to a special series or article collection?	No
**Experimental design and statistics**<br><br>Full details of the experimental design and statistical methods used should be given in the Methods section, as detailed in our Minimum Standards Reporting Checklist. Information essential to interpreting the data presented should be made available in the figure legends.<br><br>Have you included all the information requested in your manuscript?	Yes
**Resources**<br><br>A description of all resources used, including antibodies, cell lines, animals and software tools, with enough information to allow them to be uniquely	Yes

identified, should be included in the Methods section. Authors are strongly encouraged to cite Research Resource Identifiers (RRIDs) for antibodies, model organisms and tools, where possible.<br><br>Have you included the information requested as detailed in our Minimum Standards Reporting Checklist?	
**Availability of data and materials**<br><br>All datasets and code on which the conclusions of the paper rely must be either included in your submission or deposited in publicly available repositories (where available and ethically appropriate), referencing such data using a unique identifier in the references and in the "Availability of Data and Materials" section of your manuscript.<br><br>Have you have met the above requirement as detailed in our Minimum Standards Reporting Checklist?	Yes

Manuscript

Click here to access/download;Manuscript;GERONIMO_Giga_Science_for_

GERONIMO: A Tool for Systematic Retrieval of Structural RNAs in Broad Evolutionary Context

AUTHORS

Agata M. Kilar[1,2]*, Petr Fajkus[1,3] and Jiří Fajkus[1,2,3]*

[1] Mendel Centre for Plant Genomics and Proteomics, CEITEC Masaryk University, Brno CZ-62500, Czech Republic

[2] Laboratory of Functional Genomics and Proteomics, NCBR, Faculty of Science, Masaryk University, Brno CZ-61137, Czech Republic

[3] Department of Cell Biology and Radiobiology, Institute of Biophysics of the Czech Academy of Sciences, Brno CZ-61265, Czech Republic

ORCIDS: Agata Magdalena Kilar [0000-0003-3876-6581]; Petr Fajkus [0000-0001-8891-7574]; Jiri Fajkus [0000-0002-3112-1716]

* To whom correspondence should be addressed. Tel: +420 549 492 139; Email: agata.kilar@ceitec.muni.cz

Correspondence may also be addressed to Jiří Fajkus. Tel: +420 549 494 003; Email: fajkus@sci.muni.cz

ABSTRACT

Background

While web-based tools such as BLAST have made identifying conserved gene homologs appear easy, genes with variable sequences pose significant challenges. Functionally

important non-coding RNAs often show low sequence conservation due to genetic variations, including insertions and deletions. Rather than conserved sequences, these RNAs possess highly conserved structural features across a broad phylogenetic range. Such features can be identified using the covariance models approach, which combines sequence alignment with a secondary RNA structure consensus. However, running standard implementation of that approach (Infernal) requires advanced bioinformatics knowledge compared to user-friendly web services like BLAST. The issue is partially addressed by RNAcentral, which can be used to search for homologs across a broad range of ncRNA sequence collections from diverse organisms but not across the genome assemblies.

Results

Here, we present GERONIMO, which conducts evolutionary searches across hundreds of genomes in a fully-automated way. It provides results extended with taxonomy context, as summary tables and visualisations to facilitate analysis for user convenience. Additionally, GERONIMO supplements homologous sequences with genomic regions to analyse promoter motifs or gene collinearity, enhancing the validation of results.

Conclusion

GERONIMO, built using Snakemake, has undergone extensive testing on hundreds of genomes, establishing itself as a valuable tool in the identification of non-coding RNA homologs across diverse taxonomic groups. Consequently, GERONIMO facilitates the investigation of the evolutionary patterns of functionally significant ncRNA players, whose understanding has previously been limited to individual organisms and close relatives.

KEYWORDS

sequence homology searches; evolution; high-throughput pipeline; snakemake

INTRODUCTION

The characterisation of non-coding RNAs (ncRNAs) has been an ongoing and exciting area of research since the 1950s. Research has shown that ncRNAs play a vital role in various cellular processes, such as providing a decoding scaffold for transfer RNA (tRNA) (1) and building components of the translation machinery complex, such as ribosomal RNAs (rRNAs) (2). Additionally, small nuclear RNAs (snRNAs) are involved in splicing events as constituent components of the spliceosome, examples of these include U1, U2, U4, U5, and U6 [reviewed in (3)]. Subsequently, the roles of ncRNAs have been found to extend beyond translation, ribosomal activities and splicing events, encompassing diverse functions [reviewed in (4)]. Despite much progress in understanding the role and function of ncRNAs, there is still much to discover regarding new ncRNA families and their functions (5).

Classes of ncRNA significantly vary in terms of sequence length, functions, biological occurrence, structure and distribution across species (6). A notable example of ncRNA with a highly heterogeneous sequence is telomerase RNA (TR), which acts as a template for the extension of chromosome ends by the telomerase complex. The lengths of TRs vary considerably, ranging from 159 nucleotides in *Tetrahymena* to around 1 200 nucleotides in yeast, *Saccharomyces cerevisiae* (7). The extremely high diversity among TRs in terms of their lengths, sequences and biosynthesis pathways considerably complicates their identification. Consequently, TRs have been elusive in the plant kingdom for over 30 years compared to those in other organisms, such as humans or yeast (8, 9).

When performing sequence similarity searches across different species, whether evolutionarily related or not, BLAST is a popular tool and is often the first choice due to its

widespread use (10). However, BLAST is limited in identifying novel ncRNAs, as they differ from protein-coding genes and do not necessarily maintain a preserved reading frame. Thus, ncRNAs often contain small insertions or deletions that disrupt the exact seed match on which BLAST search relies. Moreover, many ncRNAs are often too short for an effective BLAST search. The limitations of sequence-homology search methods for identifying novel ncRNA sequences have been addressed by the emergence of advanced computational methods such as Infernal (11). Infernal generates probabilistic profiles of aligned ncRNA sequences and their secondary structure consensus, known as covariance models. A statistically significant covariation (correlated variation) suggests that two nucleotides in the alignment form a base pair that is important to the function of the RNA because there has been selective pressure to maintain it through compensatory base pair substitutions. A homology search performed with Infernal produced results as good as a provided covariance model. Based on this, optimisation strategies for alignments composition were proposed (12). Furthermore, Infernal has enabled the creation of the Rfam database, which contains over 3 400 reliable alignments for structural RNA families (13), and allows for the identification of homologous ncRNA sequences in related species with the aid of many pre-calculated covariance models.

In cases where existing covariance models cannot initiate homology sequence searches, it is necessary to create a new set of alignments to identify novel ncRNAs. In this process, knowledge of RNA secondary structure can be particularly helpful. Advances in affordable and precise RNA sequencing methods have significantly improved our understanding of RNA function (14). However, a "sequence-structure gap" has emerged, where many RNA molecules have been sequenced, but their structures remain unknown. To address this gap, computational tools have been proposed that aim to predict RNA secondary structure. These

tools include those based on minimum free-energy calculations, such as RNAstructure or RNAfold (15, 16), as well as those that integrate thermodynamic parameters with comparative analysis based on alignments, such as RNAalifold or Turbofold II (17, 18). The main limitations of these methods are accumulating errors in energy calculations and the tendency to "overfold" RNA structures (19). Several machine-learning methods are now being utilised to explore RNA structure (19). However, their learning process is primarily based on existing databases of RNA structure and may be biased toward certain RNA types (e.g., those used in the small training set during the learning process). Moreover, their learning process often lacks biophysical or evolutionary significance, making generalising across different RNA families challenging (20).

RNAcentral, a web-based platform, has improved the accessibility of homology searches (21). This is primarily due to its ability to allow for searches across a vast array of ncRNA databases simultaneously, using simple fasta queries. However, RNAcentral is not equipped to facilitate searches within whole genome data. In addition to this limitation, the platform also poses other challenges that can render the analysis process cumbersome. One of the principal obstacles is the indistinct evolutionary context, which necessitates time-consuming analysis of results. Often, among the vast number of results returned, only the name of the organism is given, without any information regarding the broader taxonomy, making it difficult to draw meaningful conclusions. Another challenge is the difficulty in manipulating the analysis range. The search can return an overwhelming number of results, which can be unhelpful, especially when looking for homologies in specific evolutionary lineages, particularly in the absence of a taxonomic context. The search results are presented as a text file containing the aligned sequences, with limited options for data filtering. One possible approach is to sort the results based on the similarity or significance level between the query and the targeted

sequence. However, this process can be time-consuming and requires manual inspection of the results. Lastly, lacking information on the neighbouring genomic region can hinder the validation process. Access to the genomic region around the homologous sequence could enable researchers to search for specific DNA motifs, for example, within the promoter region.

In this work, we present GERONIMO (GEnomic RNA hOmology aNd evolutIonary MOdeling), a bioinformatics pipeline that uses the Snakemake framework to conduct high-throughput homology searches of ncRNA genes using covariance models on any evolutionary scale. GERONIMO allows users to specify *what to search for* by providing a covariance model or multiple alignments in Stockholm format and *where to search* by defining a targeted database that can be easily set up at NCBI's database service and range in scale from order through family, clade to phylum or kingdom. The pipeline can be run with one command, making it easy to use without additional user intervention. After the homology search is complete, GERONIMO generates comprehensive and accessible tables that present all essential information regarding the query and target sequence similarity levels. These tables are enriched with a broad taxonomy context, which enables effective data filtering and minimises false-positive results through the assessment of evolutionary relationships. Furthermore, GERONIMO provides visual representations of the table results, which makes it easier to draw holistic conclusions and refine novel covariance models. In addition to these advancements, GERONIMO provides each significant candidate sequence with extended genomic regions, which might be searchable for specific DNA motifs, becoming handy in results validation. This makes GERONIMO a recipe for conducting routine homology searches on any taxonomy scale supported with results overview, enabling the novel ncRNAs exploration more accessible with evolutionary context.

IMPLEMENTATION

Pipeline overview. GERONIMO (RRID: SCR_023899) is a high-throughput bioinformatics pipeline that uses covariance models based on sequence alignment and secondary structure consensus to search for unknown genetic elements. It is built with Snakemake, a reproducible workflow management tool that operates on multiple computational platforms, like Windows 10 and Ubuntu. The pipeline assesses dependencies during its initial run to ensure compatibility with various platforms.

One major advantage of GERONIMO is its high level of parallelisation, which accelerates the analysis process by conducting simultaneous analyses of single genomes and covariance models. With access to 24 CPUs, for example, GERONIMO can analyse at least 6 genomes simultaneously. Snakemake's parallelisation module optimises the distribution of computational resources among all pipeline components, with most components requiring only 1 CPU.

The schematic view of GERONIMO is shown in Figure 1.

Inputs characterisation. GERONIMO accepts an NCBI database query as input, which allows for identifying the genomes used in the analysis. The database query can be easily modified at NCBI to ensure control over the desired dataset.

GERONIMO requires a covariance model to conduct evolutionary searches across the genome database. The covariance model can be obtained from the *Rfam* database (13) or built with GERONIMO using multiple sequence alignments and a consensus structure. These alignments can be generated through *RNAalifold* web services (17).

Database collection. The specified database query provided by the user is used to download the relevant genomes from the *NCBI Assembly database* (22). The *Entrez* (NCBI) tool (23) is used to generate a list of all genomes identified by the database query, which is exported to the "list_of_genomes.txt" file in the main GERONIMO folder. The links to the specific genomes are then sent to the *rsync* BASH utility, which downloads each genome and checks its integrity using the *md5 hash*. The downloaded genomes are in *.fna.gz* format. Thus, they must be unzipped for further analysis using the *gunzip* BASH utility.

Covariance model preparation (optional). GERONIMO allows creating a custom model using multiple sequence alignments with a secondary consensus structure in *.stk* format. The model is built using the incorporated Infernal module and the *cmbuilt* tool, followed by calibration with the *cmcalibrate* tool (11). To optimise the analysis flow, the user can allocate the desired number of CPUs for the calibration process in the config.yaml file, considering its resource-intensive nature.

Evolutionary search. The *cmsearch* (*Infernal)* tool conducts the search by comparing the covariance model to the genome sequence to identify similarities. Detailed information on the operation and output structures of *Infernal* can be found in the tool manual (11).

Infernal produces three output files in space-delimited text format, which can be found in the *GERONIMO/results/infernal_raw* folder, organised by model and specific genome. The results from the evolutionary search are categorised by *Infernal* based on the statistical significance of the level of similarity - *hit*. A hit is considered significant when its e-value is less than 0.01 and is marked with a "!" symbol. A value between 0.01 and 10 is regarded as potentially compelling finding and is marked with a "?" symbol. GERONIMO adheres to this

convention throughout the analysis, labelling significant hits as "HIT" and potential findings as "MAYBE". Additionally, any value greater than 10 is classified as a "NO HIT" by GERONIMO.

Reading Infernal results. The output structure of *Infernal's* files makes it challenging to concatenate the overall results, especially when conducting extensive analysis with multiple models and hundreds of genomes. To address this issue, a custom script was developed to reformat the output structure and present it in a more manageable table format. The script written in *R* (24), using the *tidyverse* package (25), handles the space-delimited nature of *Infernal's* output and converts it to an accessible *.csv* format.

Attaching taxonomy context. One limitation of working with genome assemblies downloaded from the NCBI database is that they are often described only with a GCA id, which can complicate results analysis and lack informative context. GERONIMO addresses this issue by providing a broad taxonomy context by extracting taxonomic information such as the organism name, family, order, class, and phylum. This is achieved using a custom script in *R* (24) that utilises the *rentrez* utilities (26) to extract taxonomy information from the *NCBI taxonomy database* based on the unique GCA id. The taxonomy information is then restructured into a table format, allowing it to be easily joined with the processed *Infernal* results. The individual taxonomy information can be found in the *GERONIMO/taxonomy folder*, named with the GCA id.

Extending the genomic region. GERONIMO offers extended genomic context for each significant hit by extracting the upstream and downstream region using the *blastcmd* tool preceded by the *makeblastdb* tool, both of which are part of the BLAST+ package (27). This functionality allows searching of specific DNA motifs whose presence or absence can aid in hit validation.

If a significant hit is identified in the genome with the covariance model, the coordinates of the hit are adjusted by adding or subtracting a specified length of the extracted region, depending on the location of the hit relative to the forward or reverse strand. This length can be specified in the config.yaml file under the *extract_genomic_region-length* parameter. We tested using a distance of approximately 200 nucleotides in all runs. It is important to note that genome assemblies are composed of contigs, and in some cases, the hit may be located near the beginning or end of a contig. If the region of interest exceeds the contig range, the upstream sequence cannot be extracted and requires manual curation.

Results summarisation. In the final module of GERONIMO, all individual partial results are concatenated into the *summary_table.xlsx* file, which is the main output of the pipeline. Joining the results is achieved using the *cat* BASH utility. The aggregated results are then passed to a custom *R* script which adds column names and arranges the results into a widely used *excel* table format.

To facilitate the analysis of the results, a separate "summary_table_models.xlsx" table is generated, which separates the pipeline results into spreadsheets according to the covariance models applied. This feature proves especially useful when evaluating the performance of multiple models in a single run.

Based on the summary table, GERONIMO produces summary visualisations in two plots. The first plot is a bar chart showing the number of genomes per family in which at least one significant HIT was found. The second plot presents the significance of the HITs in a heatmap, where all individual genomes are grouped into taxonomy families. The value represents the most significant HIT from each particular genome.

GERONIMO APPLICABILITY

The tool was designed to address the limitations of commonly used tools for performing sequence homology searches in an evolutionary context across multiple taxonomy levels, ranging from families to kingdoms. The tool was developed in collaboration with biology experts to ensure applicability and can be easily used with a single command line. The GERONIMO website, hosted on GitHub, includes comprehensive guidelines on tool requirements, installation, usage, and potential pitfalls described in the "Questions and Answers" section.

Standard analysis. GERONIMO is particularly useful in analyses that require testing multiple covariance models, as it provides parallelisation utilities and visual output, facilitating a better interpretation of results On a local machine equipped with an Intel(R) Core(TM) i5-10400 CPU 2.90GHz and 16Gb RAM, a standard GERONIMO run that includes 8 covariance models of highly-conserved gene families, such as snRNAs, with around 50 genome assemblies of the Rhodophyta phylum, takes approximately 2-3 hours (Example analysis available on figshare: 10.6084/m9.figshare.22266430).

Expanding the evolutionary context. By providing a new database query, the scope of the analysis can be broadened. GERONIMO will generate additional results for previously used covariance models, integrate them with earlier results, and present updated summary tables and visualisations. This is an efficient process as genomes that have already been downloaded do not need to be re-downloaded; only newly added genomes will be obtained from the updated database query. This feature is important in reducing the overall time required for complex homology searches and allowing for them to be easily updated with rapidly expanding newly available data.

Incorporating different types of models. GERONIMO offers a significant advantage in that it enables easy integration of new models into a pre-existing analysis. The tool will perform additional homology searches and combine them with previous analyses using the same database, thereby saving time and resources as existing results are not recalculated. This feature makes GERONIMO an efficient solution for adding and comparing new models.

Model training. GERONIMO offers the capability to create novel covariance models and optimise existing ones. The tool provides the option to build new covariance models and test their performance compared to original models, including situations where no covariance models are available in the Rfam database. This feature allows researchers to create and fine-tune models to meet their specific research needs.

TESTING

Validation strategy

The performance of GERONIMO was validated by comparing it to the widely used BLASTn tool. The transcribed region of *Arabidopsis thaliana* telomerase RNA (AtTR) was used as the query sequence for the BLASTn search, performed with standard parameters. Meanwhile, an alignment file with a secondary structure consensus was created from the same sequence using the RNAalifold web services (17). The input files used in the comparison are detailed in Supplemental material 1. As the dataset, we utilised representative genome assemblies of Tracheophytes available in the NCBI database (22).

Results of GERONIMO with BLASTn and RNAcentral comparision

GERONIMO's performance in homology sequence searches was compared to that of available services such as BLASTn and RNAcentral. The TR gene of *Arabidopsis thaliana* from

the Brassicales order was our search query and we selected 1101 representative genome assemblies from Tracheophytes available in the NCBI database as our reference target database. The comparison strategy is presented in Figure 2A and the results are available in Supplementary Table S1.

BLASTn identified similar sequences in approximately 5% of the targeted genomes. Specifically, it provided homologous sequences in 55% of genome assemblies belonging to the same order as the query (Brassicales). However, it failed to detect significant homologs even in closely related orders, such as Malvales or Sapindales (see Figure 2B).

The homology search conducted using RNAcentral produced a large number of results. However, these were limited solely to pre-existing annotated ncRNA collections. The search identified 38 significant sequences within the Brassicales order from 27 unique organisms, of which 24 were classified as TR. In addition, RNAcentral detected 352 similar sequences in 100 unique organisms within Tracheophyta, of which 46 were identified as TR, and the majority were classified as long ncRNA. The results were downloaded in FASTA format and manually filtered to account for evolutionary context. However, RNAcentral did not provide the secondary structure for the given query and reported that it did not match any Rfam family.

GERONIMO detected homologous sequences in 97% of the Brassicales genomes tested and identified 29 TRs in the neighbouring Sapindales order and two TRs in the Malvales order (Figure 2B). Additionally, GERONIMO detected 306 TRs in the Superrosids clade and 107 TRs in the large Superasterids clade. Notably, without any optimisation, GERONIMO could detect homologous sequences across distant clades, including basal eudicots and even monocots. To confirm the reliability of the GERONIMO results, we analysed the presence of promoter-specific motifs in the extended upstream genomic region.

Overall, all the methods tested facilitate the sorting and validation of hits based on their significance, as measured by the e-value. However, when dealing with evolutionary highly divergent ncRNAs (e.g., TRs), applying a significance threshold often results in either no hits or hits with controversial homology (i.e., high e-value). Given the challenges posed by the low conservation of ncRNAs, leveraging the surrounding genomic context offered by GERONIMO can be particularly useful in validating hits. A good example of this validation is the analysis of conserved promoter elements or gene collinearity, which may be evolutionarily more conserved than the ncRNA sequence itself.

DISCUSSION

Discovering novel ncRNA sequences through evolutionary homology might seem straightforward initially, then turn out to be challenging due to the relatively short length of ncRNAs, often 100 nucleotides or less and the lack of sequence conservation (28). Although comparative data analyses can be performed among closely related species (using BLASTn) or across the range of databases (using RNAcentral), they often lack intuitive outputs that facilitate drawing conclusions. While BLAST provides simplified taxonomic overviews, it is not well-suited for ncRNA searches, which limits the taxonomic range that can be explored. Nevertheless, BLAST can still be helpful for preliminary searches due to its user-friendly interface and ease of use. The RNAcentral Consortium is a crucial advancement in ncRNA research. It offers a consolidated platform that allows access to data from several independent ncRNA resources, including Rfam, NCBI, and ENA. The platform also supports the use of various homology search tools like nhammer, which enhances the identification of ncRNAs (21). Additionally, it provides complementary tools like R2DT, which generates secondary structure diagrams, thus improving the process of studying ncRNAs (29).

The discovery of novel ncRNAs relies on evidence of evolutionary conservation of RNA sequence and structure. Currently, structural prediction alone is insufficient evidence of a conserved structure and false discoveries can arise due to artifacts introduced by misalignments in the analysis (30). Pseudogenes pose another challenge, as they exhibit sequence and structure conservation but do not *function* as ncRNAs. The presence of pseudogenes in alignments of structural ncRNAs can dilute the covariation signal, resulting in an overall increase in power but a decrease in covariation at base-paired positions (31).

Despite the challenges associated with RNA structure analysis, Infernal has been successfully used to characterise a large number of ncRNAs in the Rfam database (32). Infernal has also been applied for the identification of novel TRs in Metazoa (33) and integrated as a module in other bioinformatics tools, including MITOS for *de novo* annotation of mitochondrial genomes (34), tRNAscan for identifying tRNAs (35, 36), and FASTAptameR 2.0 for simple motif discovery (37). Recent advances in the field have enabled the combination of Infernal with R-scape and the CaCoFold algorithm, identifying 17 novel structurally conserved ncRNA candidates across five fungal genomes (38).

As Dobzhansky famously stated, "Nothing in Biology Makes Sense Except in the Light of Evolution." (39). This statement holds true for successful homology searches, as the lack of a broad evolutionary context can hinder progress. This is especially evident for independently transcribed RNAs, where the genomic context is often more conserved than the RNA sequence itself. In addition, ncRNA genes frequently reside in homologous intergenic or intronic regions, even across large evolutionary distances (12). Moreover, GERONIMO offers a unique feature that extends the homologous sequence with neighbouring genomic context, providing the opportunity to search, for example, promoter-specific motifs. This

feature can effectively exclude false-positive candidates and detect pseudogenes, thereby strengthening the analysis. The availability of information on the neighbouring genomic region around the homologous sequence supports the validity of results. GERONIMO was developed with a focus on the broad evolutionary context, building on our previous research on the characterisation of novel TRs. This effort led to the discovery of 328 novel TRs in early-diverging plants and insects, demonstrating the effectiveness of the approach (40, 41).

GERONIMO is a unique tool that enables routine searches for novel ncRNA sequences in any evolutionary context, providing quickly analysable tables and plots of the results. The table format allows for filtering based on the significance of homologous sequences and taxonomy information, which can help identify the best candidates within a given family, order, or clade. The summary visualisation of the homology search provides insight into the overall results of the analysis, such as the number of candidates identified within a given family, which can aid in identifying the best candidates for better alignment composition and ultimately lead to improved performance of covariance models.

Finally, GERONIMO is available on various platforms, including Windows 10 and Linux, and can take advantage of external services for extensive computational power.

To date, hundreds of functionally important ncRNAs have been characterized as crucial players in various biological processes, such as adaptation to stress, chromatin remodeling, and diverse developmental pathways. Nevertheless, the knowledge about these ncRNAs often remains confined to specific model organisms. It is essential to recognize that a significant portion of these finely-tuned ncRNA machineries did not emerge by chance but rather underwent millions of years of evolution. From this perspective, GERONIMO holds the potential to offer valuable insights into ncRNA precursors present in early diverged

organisms. By analyzing the evolutionary history of these molecules across diverse taxa, GERONIMO can shed light on their ancient origins and elucidate the intricate processes that have shaped their functionality over time. This information can significantly contribute to our understanding of the broader role and conservation of ncRNAs across the tree of life, enhancing our knowledge of the fundamental biological mechanisms underlying life's complexity.

AVAILABILITY OF SOURCE CODE AND REQUIREMENTS

Project name: GERONIMO

Project home page: https://github.com/amkilar/GERONIMO.git

Operating system(s): Linux, <Windows 10 (WSL)

Programming language: Python3, bash, R

Other requirements: <conda 23.3.1

License: MIT license

RRID: SCR_023899

bio.tools ID :GERONIMO

GERONIMO is also registered on workflowhub.eu [42].

Data availability:

Supporting data sets for this article are available via Figshare [43] and GigaDB [44]

FUNDING

This work was supported by International Visegrad Fund, 52210685, Agata Magdalena Kilar; Grantová Agentura České Republiky, 20-01331X, Jiří Fajkus.

AUTHOR’S CONTRIBUTIONS

Agata M. Kilar: Conceptualization, Formal analysis, Investigation, Software, Methodology, Visualization, Funding Acquisition, Writing – original draft. Petr Fajkus: Conceptualization, Investigation, Validation, Writing – review & editing. Jiří Fajkus: Conceptualization, Supervision, Writing – review & editing, Resources.

ACKNOWLEDGEMENTS

Jakub Porc and Jakub Hynšt supported setting up the snakemake pipeline for GERONIMO. Their discussions and insights were invaluable in the development of this bioinformatics tool. Computational resources were supplied by the project' e-Infrastruktura CZ' (e-INFRA CZ LM2018140) supported by the Ministry of Education, Youth and Sports of the Czech Republic. Computational resources were provided by the ELIXIR-CZ project (LM2018131), part of the international ELIXIR infrastructure. The work was created as part of the follow-up activities in the period of sustainability of the ERDF project SYMBIT, reg. no. CZ.02.1.01/0.0/0.0/15 003/0000477. Figures were created with BioRender.com.

CONFLICT OF INTEREST

The authors declare that they have no competing interests.

REFERENCES

1. Hopper,A.K. and Phizicky,E.M. (2003) tRNA transfers to the limelight. *Genes Dev.*, **17**, 162–180.

2. Sloan,K.E., Warda,A.S., Sharma,S., Entian,K.-D., Lafontaine,D.L.J. and Bohnsack,M.T. (2017) Tuning the ribosome: The influence of rRNA modification on eukaryotic ribosome biogenesis and function. *RNA Biol.*, **14**, 1138–1152.

3. Matera,A.G., Terns,R.M. and Terns,M.P. (2007) Non-coding RNAs: lessons from the small nuclear and small nucleolar RNAs. *Nat. Rev. Mol. Cell Biol.*, **8**, 209–220.

4. Cech,T.R. and Steitz,J.A. (2014) The Noncoding RNA Revolution—Trashing Old Rules to Forge New Ones. *Cell*, **157**, 77–94.

5. Decoding noncoding RNAs (2022) *Nat. Methods*, **19**, 1147–1148.

6. Lee,H., Zhang,Z. and Krause,H.M. (2019) Long Noncoding RNAs and Repetitive Elements: Junk or Intimate Evolutionary Partners? *Trends Genet. TIG*, **35**, 892–902.

7. Singer,M.S. and Gottschling,D.E. (1994) TLC1: Template RNA Component of Saccharomyces cerevisiae Telomerase. *Science*, **266**, 404–409.

8. Richards,E.J. and Ausubel,F.M. (1988) Isolation of a higher eukaryotic telomere from Arabidopsis thaliana. *Cell*, **53**, 127–136.

9. Fajkus,P., Peška,V., Závodník,M., Fojtová,M., Fulnečková,J., Dobias,Š., Kilar,A., Dvořáčková,M., Zachová,D., Nečasová,I., *et al.* (2019) Telomerase RNAs in land plants. *Nucleic Acids Res.*, **47**, 9842–9856.

10. McGinnis,S. and Madden,T.L. (2004) BLAST: at the core of a powerful and diverse set of sequence analysis tools. *Nucleic Acids Res.*, **32**, W20–W25.

11. Nawrocki,E.P. and Eddy,S.R. (2013) Infernal 1.1: 100-fold faster RNA homology searches. *Bioinformatics*, **29**, 2933–2935.

12. Barquist,L., Burge,S.W. and Gardner,P.P. (2016) Studying RNA Homology and Conservation with Infernal: From Single Sequences to RNA Families. *Curr. Protoc. Bioinforma.*, **54**, 12.13.1-12.13.25.

13. Kalvari,I., Nawrocki,E.P., Ontiveros-Palacios,N., Argasinska,J., Lamkiewicz,K., Marz,M., Griffiths-Jones,S., Toffano-Nioche,C., Gautheret,D., Weinberg,Z., *et al.* (2021) Rfam 14: expanded coverage of metagenomic, viral and microRNA families. *Nucleic Acids Res.*, **49**, D192–D200.

14. Stark,R., Grzelak,M. and Hadfield,J. (2019) RNA sequencing: the teenage years. *Nat. Rev. Genet.*, **20**, 631–656.

15. Reuter,J.S. and Mathews,D.H. (2010) RNAstructure: software for RNA secondary structure prediction and analysis. *BMC Bioinformatics*, **11**, 129.

16. Lorenz,R., Bernhart,S.H., Höner Zu Siederdissen,C., Tafer,H., Flamm,C., Stadler,P.F. and Hofacker,I.L. (2011) ViennaRNA Package 2.0. *Algorithms Mol. Biol. AMB*, **6**, 26.

17. Bernhart,S.H., Hofacker,I.L., Will,S., Gruber,A.R. and Stadler,P.F. (2008) RNAalifold: improved consensus structure prediction for RNA alignments. *BMC Bioinformatics*, **9**, 474.

18. Tan,Z., Fu,Y., Sharma,G. and Mathews,D.H. (2017) TurboFold II: RNA structural alignment and secondary structure prediction informed by multiple homologs. *Nucleic Acids Res.*, **45**, 11570–11581.

19. Zhang,J., Fei,Y., Sun,L. and Zhang,Q.C. (2022) Advances and opportunities in RNA structure experimental determination and computational modeling. *Nat. Methods*, **19**, 1193–1207.

20. Szikszai,M., Wise,M., Datta,A., Ward,M. and Mathews,D.H. (2022) Deep learning models for RNA secondary structure prediction (probably) do not generalize across families. *Bioinforma. Oxf. Engl.*, **38**, 3892–3899.

21. The RNAcentral Consortium (2019) RNAcentral: a hub of information for non-coding RNA sequences. *Nucleic Acids Res.*, **47**, D221–D229.

22. Kitts,P.A., Church,D.M., Thibaud-Nissen,F., Choi,J., Hem,V., Sapojnikov,V., Smith,R.G., Tatusova,T., Xiang,C., Zherikov,A., *et al.* (2016) Assembly: a resource for assembled genomes at NCBI. *Nucleic Acids Res.*, **44**, D73–D80.

23. Gibney,G. and Baxevanis,A.D. (2011) Searching NCBI Databases Using Entrez. *Curr. Protoc. Bioinforma.*, **34**, 1.3.1-1.3.25.

24. The R Project for Statistical Computing. https://www.r-project.org/index.html

25. Wickham,H., Averick,M., Bryan,J., Chang,W., McGowan,L.D., François,R., Grolemund,G., Hayes,A., Henry,L., Hester,J., *et al.* (2019) Welcome to the Tidyverse. *J. Open Source Softw.*, **4**, 1686.

26. Winter,D.,J. (2017) rentrez: An R package for the NCBI eUtils API. *R J.*, **9**, 520.

27. Camacho,C., Coulouris,G., Avagyan,V., Ma,N., Papadopoulos,J., Bealer,K. and Madden,T.L. (2009) BLAST+: architecture and applications. *BMC Bioinformatics*, **10**, 421.

28. Menzel,P., Gorodkin,J. and Stadler,P.F. (2009) The tedious task of finding homologous noncoding RNA genes. *RNA*, **15**, 2075–2082.

29. Sweeney,B.A., Hoksza,D., Nawrocki,E.P., Ribas,C.E., Madeira,F., Cannone,J.J., Gutell,R., Maddala,A., Meade,C.D., Williams,L.D., *et al.* (2021) R2DT is a framework for predicting and visualising RNA secondary structure using templates. *Nat. Commun.*, **12**, 3494.

30. Rivas,E. (2021) Evolutionary conservation of RNA sequence and structure. *WIREs RNA*, **12**.

31. Gao,W., Yang,A. and Rivas,E. (2022) Thirteen dubious ways to detect conserved structural RNAs. *IUBMB Life*, **n/a**.

32. Griffiths-Jones,S. (2004) Rfam: annotating non-coding RNAs in complete genomes. *Nucleic Acids Res.*, **33**, D121–D124.

33. Logeswaran,D., Li,Y., Podlevsky,J.D. and Chen,J.J.-L. (2021) Monophyletic Origin and Divergent Evolution of Animal Telomerase RNA. *Mol. Biol. Evol.*, **38**, 215–228.

34. Bernt,M., Donath,A., Jühling,F., Externbrink,F., Florentz,C., Fritzsch,G., Pütz,J., Middendorf,M. and Stadler,P.F. (2013) MITOS: Improved de novo metazoan mitochondrial genome annotation. *Mol. Phylogenet. Evol.*, **69**, 313–319.

35. Lowe,T.M. and Eddy,S.R. (1997) tRNAscan-SE: A Program for Improved Detection of Transfer RNA Genes in Genomic Sequence. *Nucleic Acids Res.*, **25**, 955–964.

36. Lowe,T.M. and Chan,P.P. (2016) tRNAscan-SE On-line: integrating search and context for analysis of transfer RNA genes. *Nucleic Acids Res.*, **44**, W54–W57.

37. Kramer,S.T., Gruenke,P.R., Alam,K.K., Xu,D. and Burke,D.H. (2022) FASTAptameR 2.0: A web tool for combinatorial sequence selections. *Mol. Ther. - Nucleic Acids*, **29**, 862–870.

38. Gao,W., Jones,T.A. and Rivas,E. (2021) Discovery of 17 conserved structural RNAs in fungi. *Nucleic Acids Res.*, **49**, 6128–6143.

39. Dobzhansky,T. (1973) Nothing in Biology Makes Sense except in the Light of Evolution. *Am. Biol. Teach.*, **35**, 125–129.

40. Fajkus,P., Kilar,A., Nelson,A.D.L., Holá,M., Peška,V., Goffová,I., Fojtová,M., Zachová,D., Fulnečková,J. and Fajkus,J. (2021) Evolution of plant telomerase RNAs: farther to the past, deeper to the roots. *Nucleic Acids Res.*, **49**, 7680–7694.

41. Fajkus,P., Adámik,M., Nelson,A.D.L., Kilar,A.M., Franek,M., Bubeník,M., Frydrychová,R.Č., Votavová,A., Sýkorová,E., Fajkus,J., *et al.* (2023) Telomerase RNA in Hymenoptera (Insecta) switched to plant/ciliate-like biogenesis. *Nucleic Acids Res.*, **51**, 420–433.

42. Kilar, A. et al *GERONIMO*. WorkflowHub. 2023 https://doi.org/10.48546/workflowhub.workflow.547.1

43. Kilar A, Fajkus P, Fajkus J. GERONIMO: GEnomic RNA hOmology aNd evolutIonary MOdeling. figshare repository. 2023. https://doi.org/10.6084/m9.figshare.22266430.v2

44. Kilar AM; Fajkus P; Fajkus J. Supporting data for "GERONIMO: A Tool for Systematic Retrieval of Structural RNAs in Broad Evolutionary Context" GigaScience Database. 2023. http://dx.doi.org/10.5524/102438

TABLE AND FIGURES LEGENDS

Figure 1. The GERONIMO workflow representing the pipeline components.

Figure 2. Overview of the strategy for comparing the performance of GERONIMO, RNAcentral and BLASTn and their results. A) The comparison process involved using the Arabidopsis thaliana Telomerase RNA (AtTR) sequence as a query in a targeted database of 1101 genome assemblies available at the NCBI database to find homologous candidates. The use of GERONIMO yielded results that were supported by extended genomic regions and broad taxonomic contexts. In contrast, RNAcentral only provided homologous sequences, while BLASTn offered homologous sequences with plain evolutionary context. B) A simplified phylogenetic tree of the Tracheophyta clade was used, including the extended orders of Superasterids and Superrosids, along with the number of representative genome assemblies used in the comparison. The branch lengths do not express real-time scales. The green bar represents the performance of GERONIMO, the violet bar represents the performance of RNAcentral, and the red bar represents the performance of BLASTn, with the number of genomes in which TR candidates were identified (marked with a green tick). The red cross indicates a failure to identify TR within a given order.

Figure 1 Click here to access/download;Figure;Figure 1.png

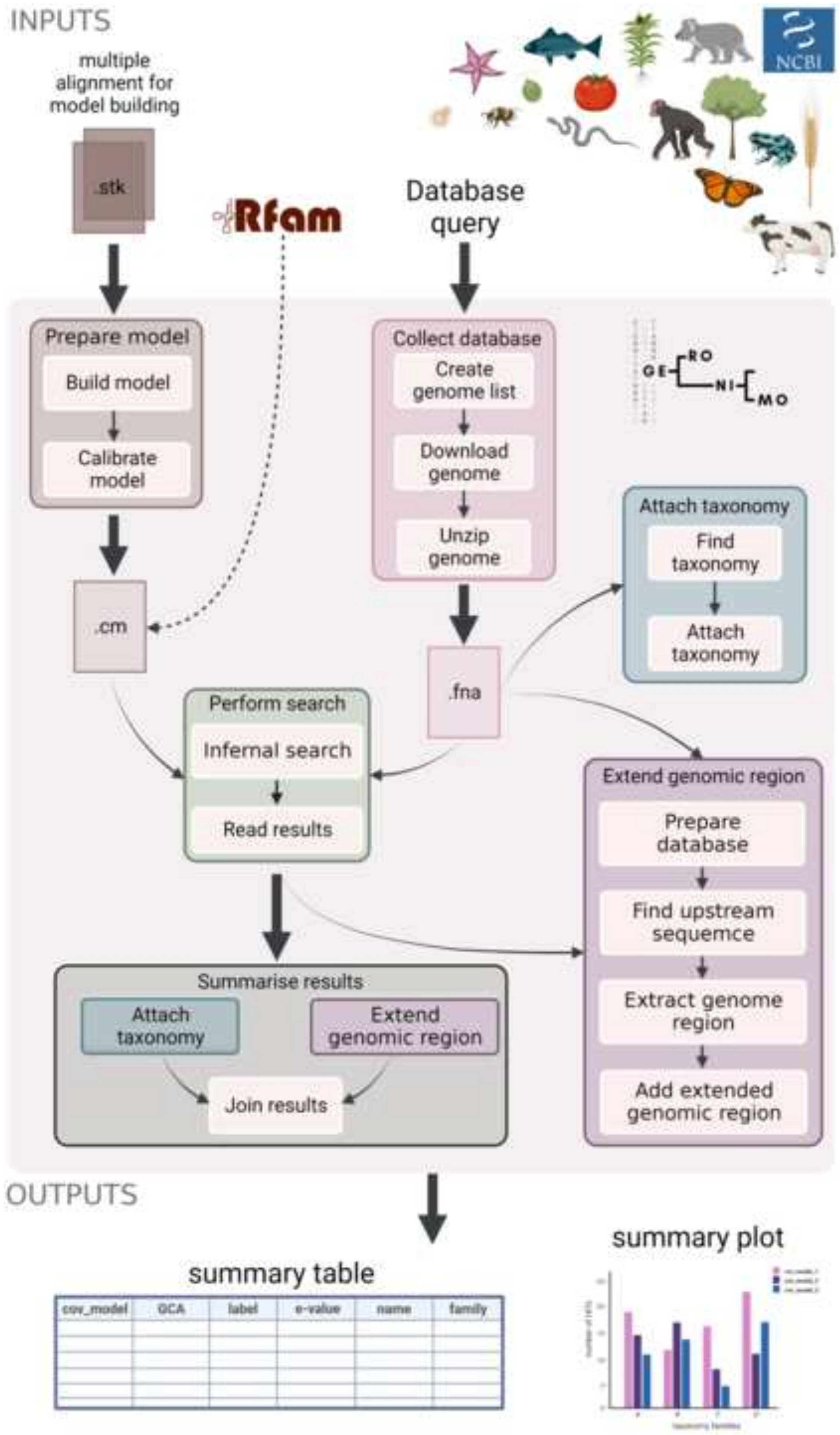

Figure 2 Click here to access/download;Figure;Figure 2.png

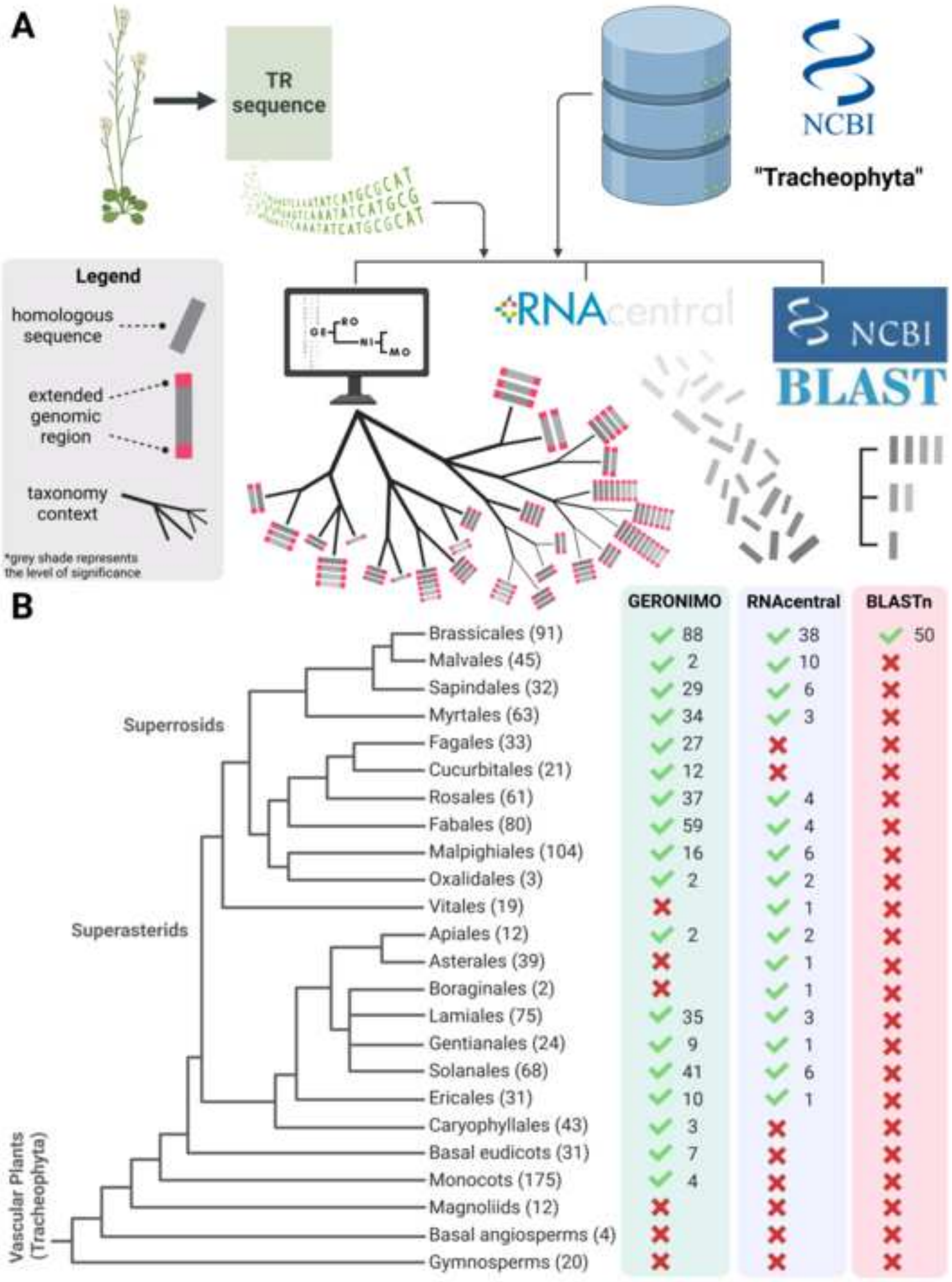

Supplementary Table S1

Click here to access/download
Supplementary Material
Supplementary Table S1.xlsx

Supplementary material 1

Click here to access/download

Supplementary Material

Supplementary material 1.docx
