# Supplementary material for: GERONIMO: A tool for systematic retrieval of structural RNAs in a broad evolutionary context
Source: Gigascience. 2023 Oct 17;12:giad080. doi: 10.1093/gigascience/giad080 (PMC10580375; doi:10.1093/gigascience/giad080)

1. AtTR query used in BLASTn:

>AB646770.1 Arabidopsis thaliana gene for long non-coding RNA

AAAGCACTTTGTCCCACATCGGAAGATAGAGATTGAAGTTGAGTTGTTTAAATGAAGAAAGGTTTGGTCA

AATAAGTTAAGGGGTGTGGGAACCTAGGAGATGAGTCTGCTTATTGATTGCTAAACCCTGAACCCTCTCA

TGTTAACTATGGGAATTAATTACTGGGGGTCTTAGGCCGGCGTTTCCCCCGAAAAATAATGAAAAAACGT

CGATGGCTACATAAGAGGCTCGTCTCCAAAAAGTAGACCAGGAGGTTGGGTTTGGTTCGTAGGTGGTTCT

GTTGAAACTAGATTAGTGTCTGCGGTTAACCGTTTCCTCGCCTTACCCTCCCACCCCCAAATATTTTGTT

TTCTATTTTTTATTTTAAGTAATGATGGTTTTTACATCAACAAATATTGA

1. Prerequisites for building the covariance model by GERONIMO:

A) .stk format file used for building the AtTR covariance model:

# STOCKHOLM 1.0

#=GF CC Generated by LocARNA 2.0.0RC8

#=GF SQ 2

Arabidopsis_thaliana_1 AGUUAAGGGGUGUGGGAACCUAGGAGAUGAGUCUGCUUAUUGAUUGCUAAACCCUGAACCCUCUCAUGUUAACUAUGGGAAUUAAUUACUGGGGGUCUUAGGCCGGCGUUUCCCCCGAAA

Arabidopsis_thaliana_2 AGUUAAGGGGUGUGGGAACCUAGGAGAUGAGUCUGCUUAUUGAUUGCUAAACCCUGAACCCUCUCAUGUUAACUAUGGGAAUUAAUUACUGGGGGUCUUAGGCCGGCGUUUCCCCCGAAA

#=GC SS_cons ......((((.((((((.....((((((((((((.(((((.....((((...(((((((((((....(((((.........)))))....)))))).))))).((((((((.........

Arabidopsis_thaliana_1 AAUAAUGAAAAAACGUCGAUGGCUACAUAAGAGGCUCGUCUCCAAAAAGUAGACCAGGAGGUUGGGUUUGGUUCGUAGGUGGUUCUGUUGAAACUAGAUUAGUGUCUGCGGUUAACCGUU

Arabidopsis_thaliana_2 AAUAAUGAAAAAACGUCGAUGGCUACAUAAGAGGCUCGUCUCCAAAAAGUAGACCAGGAGGUUGGGUUUGGUUCGUAGGUGGUUCUGUUGAAACUAGAUUAGUGUCUGCGGUUAACCGUU

#=GC SS_cons ..........)))))))).))))...)))))))))))))))))........((((....))))((((..((..((.((((((((((((.((.((((...)))).)).))))..)))))..

Arabidopsis_thaliana_1 UCCUCGCCUUACCCUCCCACCCCCAAAUAUUU

Arabidopsis_thaliana_2 UCCUCGCCUUACCCUCCCACCCCCAAAUAUUU

#=GC SS_cons .)))))))..))))))))))))))........

//

1. visualisation of secondary structure consensus:


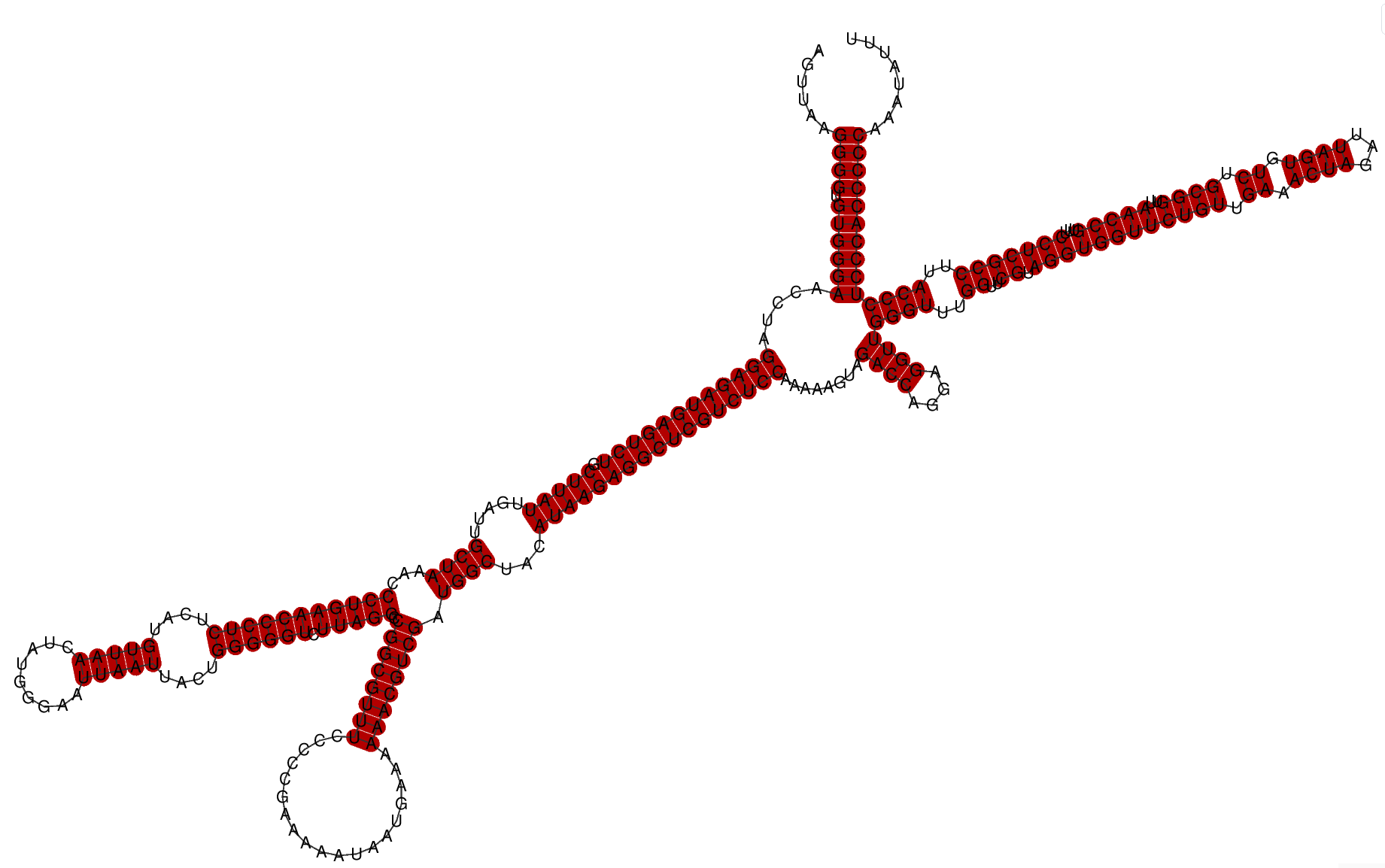

Supplement: giad080_Supplemental_Files [file giad080_supplemental_files.zip › Supplementary material 1.docx]
