# Supplementary material for: GERONIMO: A tool for systematic retrieval of structural RNAs in a broad evolutionary context
Source: Gigascience. 2023 Oct 17;12:giad080. doi: 10.1093/gigascience/giad080 (PMC10580375; doi:10.1093/gigascience/giad080)
Supplement: giad080_Response_to_Reviewer_Comments_Original_Submission [file giad080_response_to_reviewer_comments_original_submission.pdf]

Author's Response To Reviewer Comments

Dear Editor,
Thank you very much for communicating our MS to qualified reviewers. We are pleased to submit the minor revision addressing all your and reviewers' comments. Please, see below our responses:

EDITOR:

- In addition to the reviewer's reports, I'd like to mention that submissions presenting new software should discuss and compare similar, existing tools - I see you compare to BLASTn and RNAcentral, but if you are aware of any other publicly available tools that are similar or related to GERONIMO, please make sure to mention and compare them in the revised submission.

RESPONSE: Thank you for your thoughtful feedback on our manuscript. We are aware of the Evolinc II pipeline developed by A. Nelson (https://www.ncbi.nlm.nih.gov/pmc/articles/PMC5422434/), and we acknowledge its relevance as another tool aimed at identifying evolutionarily conserved ncRNAs using RNAseq data and genome assemblies. However, after carefully assessing Evolinc II, we found that the mutual comparisons of lincRNAs are BLAST-based, similar to our comparison with BLASTn. Therefore, we believe that a direct comparison with Evolinc II may not bring substantial new insights to our manuscript.

Furthermore, we are also aware that nhmmer can be employed for similar searches against genomes, but we had already implemented and utilized it in our comparison, including in the assessment against RNAcentral.

We firmly believe that the existing comparisons with RNAcentral and BLASTn sufficiently support the claims made in our submission. Nonetheless, we are grateful for your suggestion and appreciate your interest in further validation. If you have any other questions or concerns, please do not hesitate to share them with us. We are committed to improving the manuscript based on valuable feedback like yours.

- Please also carefully read our instructions for authors for the "Technical Note" article type and make sure you follow the formatting guidelines (order and headings of sections etc.)

RESPONSE: We have carefully reviewed the instructions for authors regarding the "Technical Note" article type and have made the necessary changes to ensure that our manuscript adheres to the formatting guidelines, including the correct order and headings of sections.

- In addition, please register your new software application in the bio.tools and SciCrunch.org databases to receive RRID (Research Resource Identification Initiative ID) and biotoolsID identifiers, and include these in your manuscript (in the "code availability" section). Computational workflows should be registered in workflowhub.eu and the DOIs cited in the relevant places in the manuscript. These will facilitate tracking, reproducibility and re-use of your tool.

RESPONSE: We acknowledge the importance of providing RRID and biotoolsID identifiers for our tool and ensuring that computational workflows are registered in appropriate databases like workflowhub.eu. We have taken immediate action and successfully registered GERONIMO in both bio.tools and SciCrunch.org databases, obtaining the necessary RRID and biotoolsID identifiers (SCR_023899 and biotools:GERONIMO, respectively). Additionally, we have also registered our computational workflows on workflowhub.eu and included the relevant DOIs (https://doi.org/10.48546/workflowhub.workflow.547.1) in the manuscript, as you suggested.

REVIEWER #1:
No questions or suggestions.

REVIEWER #2:

- The authors use a value of 0.05 as the E value cutoff for 'true' hits and suggest it is infernal's standard value. However, the infernal manual recommends 0.01, http://eddylab.org/infernal/Userguide.pdf page 20. Was this difference intentional?

RESPONSE: Upon carefully considering your comments, we have identified an unintentional mistake in our study regarding the e-value cutoff used for identifying 'true' hits.

You are correct in pointing out that we used a value of 0.05 as the e-value cutoff for 'true' hits, while the infernal manual recommends a more stringent value of 0.01 (http://eddylab.org/infernal/Userguide.pdf page 20). We apologize for this oversight in our initial submission.

We want to assure you that this difference in the e-value cutoff was not intentional, and we recognize the significance of using the appropriate threshold to ensure the accuracy and reliability of our findings. As soon as we became aware of this mistake through your review, we took immediate action to rectify it.

We have now revised the manuscript to reflect the correct e-value cutoff of 0.01 in all relevant sections.

- Additionally, infernal itself indicates if something is a true vs false hit using '!' or '?' in the output. Are the authors using infernal's filters or their own to select hits?

RESPONSE: In our study, we have indeed utilized infernal's '!' and '?' symbols for this purpose. However, to enhance clarity and avoid any confusion, we have translated these symbols into "HIT" and "MAYBE" in the manuscript. This modification has been made in the "Methods" section to ensure a clear representation of our approach.

We hope that this clarification addresses your concern, and we thank you for helping us improve the clarity and accuracy of our research.

- It may be necessary to use the -Z option of cmsearch to produce accurate E values. If the genome is split across several files then it is required to ensure E values are accurate. If the genome is in one file then it is not needed as cmsearch will compute it correctly. This reviewer did not examine the pipeline closely enough to determine if the genomes being searched are in a single file.

RESPONSE: In our pipeline, we perform searches on whole genomes represented as single files using Infernal. As such, we did not utilize the -Z option in the cmsearch.

It is essential to clarify that our parallelization process does not involve splitting the genome; instead, it distributes the entire genomes among the available CPUs for efficient processing.

- This reviewer isn't terribly familiar with conda environments, but the yaml files it uses appear to mention paths specific to one of the authors computers. Is this expected?

RESPONSE: You are absolutely right; we apologize for any confusion caused by the paths specified in the conda environment yaml files. Rest assured, before finalizing the manuscript, we extensively tested our software, GERONIMO, on multiple systems, not just the one with the specific path mentioned.

The results of our testing were positive, and GERONIMO performed as expected on various setups, including those without any specific paths mentioned in the yaml files. We have now made the necessary adjustments to ensure that the paths are generalized and applicable across different systems.

- There are some small cleanups that could be done to the produced figures. For example, the facet names in plot Hits_distribution_across_families, should be the model not prefixed with cov_. The green color is unclear in plots/Hits_distribution_heatmap. It is not labelled in the figure but should be. In general the figures could use some polish to be more approachable to new users.

RESPONSE: We have made the necessary adjustments to address your comments. Specifically, in the plot

"Hits_distribution_across_families," we have updated the facet names to display the model without the "cov_model_" prefix, as you suggested. Additionally, in the "Hits_distribution_heatmap" plot, we have now changed the green color to grey to enhance clarity.

Furthermore, we have taken your feedback into consideration for all the figures, and we have applied touch-ups to improve their overall presentation and make them more approachable for new users.

- The images in their very comprehensive README file are broken.

RESPONSE: We would like to express our gratitude for bringing to our attention the issue with the images in our comprehensive README file.

We are pleased to inform you that we have promptly addressed this concern and fixed the broken images. The README file now displays all the images correctly, ensuring that users can access the necessary information seamlessly.

- 'The pipeline can be run with one command line' should be 'The pipeline can be run with one command'.

RESPONSE: We acknowledge the correction you pointed out regarding the statement, "The pipeline can be run with one command line." We have now revised it to say, "The pipeline can be run with one command."
