# Supplementary material for: GERONIMO: A tool for systematic retrieval of structural RNAs in a broad evolutionary context
Source: Gigascience. 2023 Oct 17;12:giad080. doi: 10.1093/gigascience/giad080 (PMC10580375; doi:10.1093/gigascience/giad080)
Supplement: giad080_Reviewer_2_Report_Original_Submission — Blake Sweeney -- 7/10/2023 Reviewed [file giad080_reviewer_2_report_original_submission.pdf]

Reviewer Report

Title: GERONIMO: A Tool for Systematic Retrieval of Structural RNAs in Broad Evolutionary Context

Version: Original Submission **Date:** 7/10/2023

Reviewer name: Blake Sweeney

Reviewer Comments to Author:

The authors present GERMINO, a snakemake pipeline to identify novel ncRNA genes. It is based on using infernal to search a user-provided selection of genomes and models to identify regions which may contain novel ncRNAs. The tool can extract a user configurable region of the genome for further analysis. It also produces figures to help users understand the distribution of model hits. Identifying model hits in a selected set of genomes is a common problem in ncRNA studies and many groups have developed similar tooling, but those tools are not generally available.Major comments- The authors use a value of 0.05 as the E value cutoff for 'true' hits and suggest it is infernal's standard value. However, the infernal manual recommends 0.01, http://eddylab.org/infernal/Userguide.pdf page 20. Was this difference intentional? Additionally, infernal itself indicates if something is a true vs false hit using '!' or '?' in the output. Are the authors using infernal's filters or their own to select hits?- It may be necessary to use the -Z option of cmsearch to produce accurate E values. If the genome is split across several files then it is required to ensure E values are accurate. If the genome is in one file then it is not needed as cmsearch will compute it correctly. This reviewer did not examine the pipeline closely enough to determine if the genomes being searched are in a single file.Minor comments- This reviewer isn't terribly familiar with conda environments, but the yaml files it uses appear to mention paths specific to one of the authors computers. Is this expected?- There are some small cleanups that could be done to the produced figures. For example, the facet names in plot Hits_distribution_across_families, should be the model not prefixed with cov_. The green color is unclear in plots/Hits_distribution_heatmap. It is not labelled in the figure but should be. In general the figures could use some polish to be more approachable to new users.- The images in their very comprehensive README file are broken.Typos- 'The pipeline can be run with one command line' should be 'The pipeline can be run with one command'.

Level of Interest

Please indicate how interesting you found the manuscript: Choose an item.

Quality of Written English

Please indicate the quality of language in the manuscript: Choose an item.

Declaration of Competing Interests

Please complete a declaration of competing interests, considering the following questions:

- Have you in the past five years received reimbursements, fees, funding, or salary from an organisation that may in any way gain or lose financially from the publication of this manuscript, either now or in the future?
- Do you hold any stocks or shares in an organisation that may in any way gain or lose financially from the publication of this manuscript, either now or in the future?
- Do you hold or are you currently applying for any patents relating to the content of the manuscript?
- Have you received reimbursements, fees, funding, or salary from an organization that holds or has applied for patents relating to the content of the manuscript?
- Do you have any other financial competing interests?
- Do you have any non-financial competing interests in relation to this paper?

If you can answer no to all of the above, write 'I declare that I have no competing interests' below. If your reply is yes to any, please give details below.

I declare that I have no competing interests

I agree to the open peer review policy of the journal. I understand that my name will be included on my report to the authors and, if the manuscript is accepted for publication, my named report including any attachments I upload will be posted on the website along with the authors' responses. I agree for my report to be made available under an Open Access Creative Commons CC-BY license (http://creativecommons.org/licenses/by/4.0/). I understand that any comments which I do not wish to be included in my named report can be included as confidential comments to the editors, which will not be published.

Choose an item.

To further support our reviewers, we have joined with Publons, where you can gain additional credit to further highlight your hard work (see: https://publons.com/journal/530/gigascience). On publication of this paper, your review will be automatically added to Publons, you can then choose whether or not to claim your Publons credit. I understand this statement.

Yes Choose an item.
